# Genomic insights into local-scale evolution of ocular Chlamydia trachomatis strains within and between individuals in Gambian trachoma-endemic villages

**DOI:** 10.1099/mgen.0.001210

**Published:** 2024-03-06

**Authors:** Ehsan Ghasemian, Nkoyo Faal, Harry Pickering, Ansumana Sillah, Judith Breuer, Robin L. Bailey, David Mabey, Martin J. Holland

**Affiliations:** 1 Department of Clinical Research, London School of Hygiene & Tropical Medicine, London, UK; 2 Medical Research Council Unit The Gambia at London School of Hygiene and Tropical Medicine, Banjul, Gambia; 3 National Eye Health Programme, Ministry of Health, Kanifing, Gambia; 4 Division of Infection and Immunity, University College London, London, UK

**Keywords:** *Chlamydia trachomatis*, evolution, trachoma, whole-genome sequencing (WGS)

## Abstract

Trachoma, a neglected tropical disease caused by *Chlamydia trachomatis* (Ct) serovars A–C, is the leading infectious cause of blindness worldwide. Africa bears the highest burden, accounting for over 86 % of global trachoma cases. We investigated Ct serovar A (SvA) and B (SvB) whole genome sequences prior to the induction of mass antibiotic drug administration in The Gambia. Here, we explore the factors contributing to Ct strain diversification and the implications for Ct evolution within the context of ocular infection. A cohort study in 2002–2003 collected ocular swabs across nine Gambian villages during a 6 month follow-up study. To explore the genetic diversity of Ct within and between individuals, we conducted whole-genome sequencing (WGS) on a limited number (*n*=43) of Ct-positive samples with an *omc*B load ≥10 from four villages. WGS was performed using target enrichment with SureSelect and Illumina paired-end sequencing. Out of 43 WGS samples, 41 provided sufficient quality for further analysis. *omp*A analysis revealed that 11 samples had highest identity to *omp*A from strain A/HAR13 (NC_007429) and 30 had highest identity to *omp*A from strain B/Jali20 (NC_012686). While SvB genome sequences formed two distinct village-driven subclades, the heterogeneity of SvA sequences led to the formation of many individual branches within the Gambian SvA subclade. Comparing the Gambian SvA and SvB sequences with their reference strains, Ct A/HAR13 and Ct B/Jali20, indicated an single nucleotide polymorphism accumulation rate of 2.4×10^−5^ per site per year for the Gambian SvA and 1.3×10^−5^ per site per year for SvB variants (*P*<0.0001). Variant calling resulted in a total of 1371 single nucleotide variants (SNVs) with a frequency >25 % in SvA sequences, and 438 SNVs in SvB sequences. Of note, in SvA variants, highest evolutionary pressure was recorded on genes responsible for host cell modulation and intracellular survival mechanisms, whereas in SvB variants this pressure was mainly on genes essential for DNA replication/repair mechanisms and protein synthesis. A comparison of the sequences between observed separate infection events (4–20 weeks between infections) suggested that the majority of the variations accumulated in genes responsible for host–pathogen interaction such as CTA_0166 (phospholipase D-like protein), CTA_0498 (TarP) and CTA_0948 (deubiquitinase). This comparison of Ct SvA and SvB variants within a trachoma endemic population focused on their local evolutionary adaptation. We found a different variation accumulation pattern in the Gambian SvA chromosomal genes compared with SvB, hinting at the potential of Ct serovar-specific variation in diversification and evolutionary fitness. These findings may have implications for optimizing trachoma control and prevention strategies.

## Data Summary

Gambian *Chlamydia trachomatis* sequencing data in the form of fastq.gz files used in this study can be accessed from the European Nucleotide Archive (ENA) project accession PRJEB68379 (accessions ERR12330790–ERR12330830). Reference strains B/Tunis864 and B/HAR36 sequencing data and assemblies in the form of fastq.gz and fasta.gz files can be accessed from the European Nucleotide Archive (ENA) project accession PRJEB68374 (accessions ERR12253485–ERR12253486). All packages used for data analysis are linked to citations.

**Impact Statement**

*Chlamydia trachomatis* (Ct) is a globally significant pathogen. It is the leading infectious cause of blindness – a disease called trachoma. In addition, Ct causes the majority of bacterial sexually transmitted infections. Current control measures for trachoma are based on the ‘SAFE’ strategy: Surgery for trichiasis (S), Antibiotics (A), Facial cleanliness (F) and Environmental improvement (E). Whilst this strategy has achieved remarkable success, the target date for the global elimination of blinding trachoma as a public health problem has been pushed back from 2020 to 2030. Previous studies have provided evidence indicating variations in infection loads and severity among different ocular Ct serovars. However, there remains a significant knowledge gap regarding the specific genes and mechanisms responsible for these variations. We generated genetic data from two main serovars of Ct that infect human eyes, serovar A (SvA) and serovar B (SvB) variants, collected from four villages in two different administrative regions on opposing sides of the river Gambia to elucidate (i) the factors driving the diversification of ocular Ct strains; (ii) disparities in mutation frequency/accumulation profiles; (iii) selective pressures between SvA and SvB; and (iv) the dynamics of mutation accumulation within the Gambian ocular Ct-positive population over a short timeframe. Our findings suggest a different variation accumulation pattern in SvA chromosomal genes compared with SvB, hinting at the potential of Ct serovar-specific variation in diversification and evolutionary fitness. These findings may have implications for optimizing trachoma control and prevention strategies.

## Introduction

Trachoma, a neglected tropical disease, is the leading infectious cause of blindness worldwide, affecting marginalized populations in low-resource settings [1,2]. Trachoma is primarily caused by *Chlamydia trachomatis* (Ct) serovars A–C, with serovars A (SvA) and B (SvB) being the most commonly associated with ocular infection in Africa [3,4]. Worldwide trachoma is responsible for the visual impairment or blindness of about 1.9 million people [5,7]. Currently, an estimated 115.7 million people are at risk [6,7]. The highest concentrations of this neglected disease include 42 countries in Africa, the Middle East, Asia, and Central and South America, along with Australia. Africa has over 86 % of the world’s known trachoma cases [7].

There are clear disparities in tissue tropism, disease outcome and growth rates among ocular, urogenital (UGT) and lymphogranuloma venereum (LGV) strains that are attributed to key variations in virulence genes, including *omp*A, *tar*P, *pmp*s, *trp*AB, the cytotoxin locus and *inc*A, although the genomes are highly conserved and evolutionary mechanisms have only been partially explained [8,14]. Several comparative genomics investigations have contributed novel insights into the genetic diversity and evolution of Ct [10,11, 15,19]. For instance, Ct is known to undergo homologous recombination and acquire point mutations that affect tissue tropism and virulence [10,11, 15]. There is cumulative evidence that implicates a family of proteins unique to *Chlamydiae*, the polymorphic membrane proteins (Pmps), in promoting niche-specific adhesion [20,21]. A study by Gomes *et al*. [22] revealed that LGV strains carry specific amino acid substitutions in PmpB, C, D and G that distinguish them from non-LGV strains, and differences in Pmp E, F and H that segregate ocular from UGT and LGV strains. In a whole-genome sequencing (WGS) study focused on UGT Ct genotypes E and F, substantial genetic variations were identified, particularly in coding sequences related to membrane proteins such as *pmp* E and F, Type III secreted proteins (T3SS) and the cytotoxin locus, which support the assumption of higher evolutionary variability of genes involved in interactions with the host [16].

Within trachoma populations, a comparative WGS analysis of Ct strains collected from Sudanese trachoma patients [15] indicated minimal genomic diversity within this specific population. However, analysing the genome phylogeny of the 12 Ct SvA strains from the study revealed a distinctive subclade within the larger trachoma lineage, probably stemming from an evolutionary bottleneck. Notably, three genes, namely CTA_0172, CTA_0173 and CTA_0482, exhibited extensive allelic variation, suggesting that altered expression or activity of these genes may impact the growth and survival of these ocular strains [15]. Furthermore, in a study conducted by Pickering *et al*. [3] involving trachoma patients from Amhara, Ethiopia, polymorphisms near the *omp*A locus, combined with heightened *omp*A diversity, were linked to village-level trachomatous inflammation-follicular (TF) and increased Ct infection prevalence at the district level, respectively.

Previous findings by West *et al*. [23], Last *et al*. [24] and Solomon *et al*. [25] on trachoma patients suggested that the infection load might be an essential factor in the transmission of infection. Several studies on trachoma endemic communities showed that higher Ct loads were associated with trachomatous inflammation-intense (TI). These studies demonstrated a link between higher Ct loads and increasing severity of inflammation in the conjunctiva [24,26, 27]. Previously, a study by Ghasemian *et al*. [28] on Moroccan trachoma patients showed a significantly higher load of Ct in patients infected with SvB compared with those infected with SvA. However, there remains a significant knowledge gap regarding the specific genes and mechanisms responsible for variations in infection loads among different ocular Ct serovars. We generated genetic data from 11 SvA and 30 SvB variants collected from four villages in two administrative regions on opposing sides of the river Gambia to elucidate: (i) the factors driving the diversification of ocular Ct strains; (ii) disparities in mutation frequency and accumulation profiles; (iii) selective pressures between SvA and SvB; and (iv) the dynamics of mutation accumulation within the Gambian ocular Ct-positive population over a short timeframe. This study was done in the context of an investigation of Ct infection-induced immune responses and protection in trachoma [29,30], with Ct molecular diagnosis and *omp*A sequencing the priority for the extracted DNA [31,33]. Nevertheless, an opportunistic selection of samples from this prospective cohort study allowed us to analyse Ct genomes from individuals who repeatedly tested positive over the study period, and sheds light on both immediate and long-term evolutionary trends within SvA and SvB strains from The Gambia.

## Methods

### Ethics

The samples were collected and archived under the following approvals: the joint scientific and ethics committee of the Gambian Government-Medical Research Council Gambia Unit and the London School of Hygiene and Tropical Medicine (MRC SCC: 745/781; MRC SCC L2008.75; LSHTM: 535). The study was conducted in accordance with the principles of the Declaration of Helsinki. Community leaders provided verbal consent, while written informed consent was acquired from the guardians of all study participants. In this context, a signature or thumbprint was considered an acceptable form of consent. At the time of consent, archive and secondary use were included for their potential use in pathogen genotyping studies.

### Sample collection

**Fig. 1.**
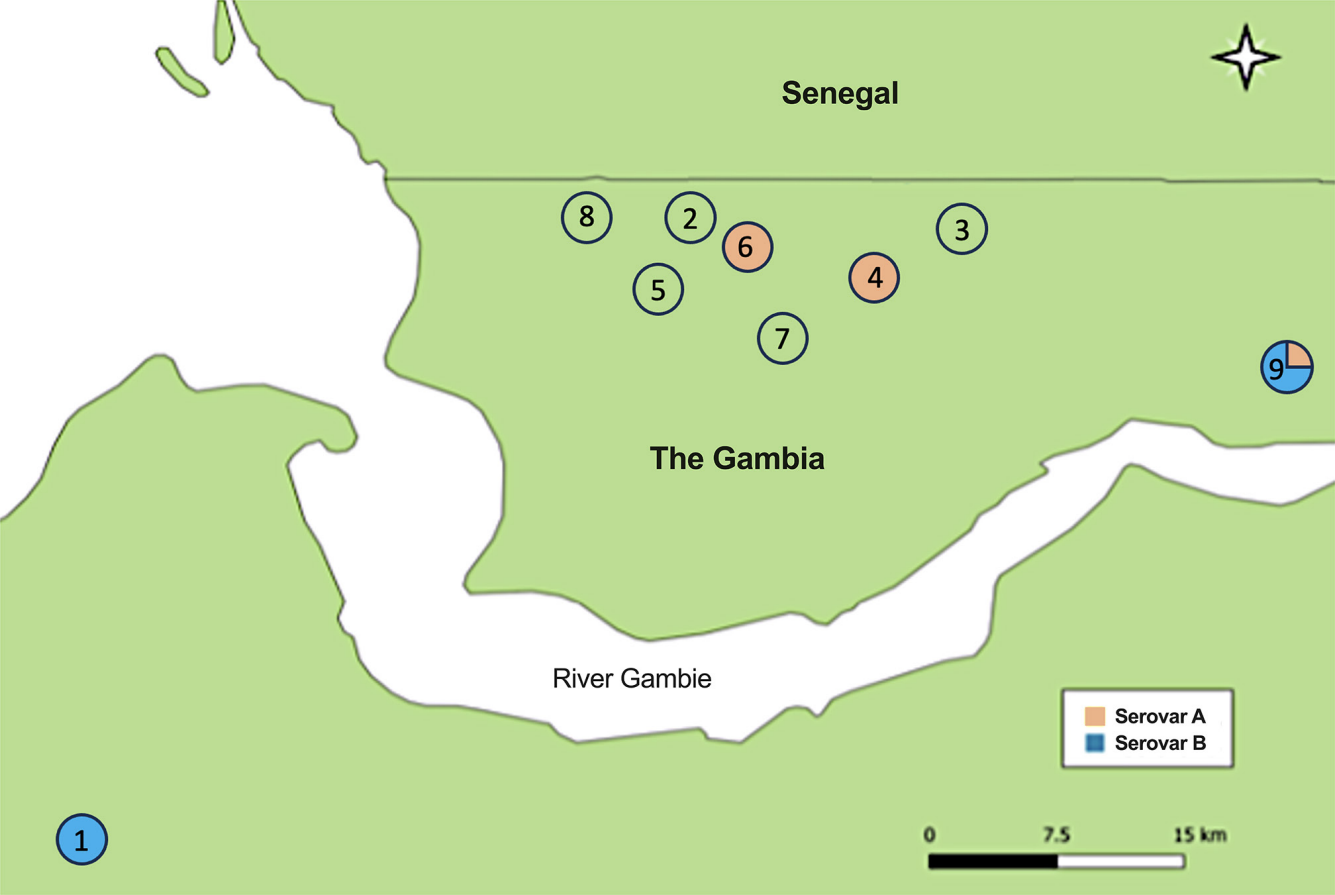
Sampling sites and geographical distribution of *Chlamydia trachomatis omp*A among the Gambian samples. In total nine villages in The Gambia were included in the sampling process from which 41 samples from villages 1, 4, 6 and 9 provided good quality whole-genome sequences that were included in this study. Red colour indicates the presence of *C. trachomatis* (Ct) serovar A in the village, and blue colour indicates the presence of Ct serovar B in the village. Empty circles depict villages that were included in the sampling process but did not provide Ct whole genomes.

For the initial screening, nine villages were chosen based on information from the Gambian National Eye Care Programme (NECP), which conducted a trachoma rapid assessment survey in the Western and North Bank Regions, identifying villages where active trachoma was approximately 20 % in school-age children. After this screen we recruited a cohort of 345 children aged 4–15 years from 31 family compounds, who were visited from 0 to 28 weeks every 2 weeks. Of note, 41 samples from 26 participants used in our WGS study originated from four villages (Fig. 1). The study was conducted before mass drug administration (MDA) for trachoma control by The Gambia’s National Eye Health Programme (NEHP). Therefore, children with intense inflammatory trachoma were treated upon diagnosis, and at the study’s end, all household members were offered oral azithromycin treatment.

Trachoma was graded by two experienced examiners whose observations are regularly validated by testing with the World Health Organization (WHO) trachoma grading slides, with an in-house slide and photograph collection and in the course of numerous field studies. Trachoma was graded using the WHO simplified grading systems for clinical signs by the same team of experienced trachoma graders [34]. Ocular swabs were taken by a single trained examiner from the everted tarsal conjunctiva of the child’s right eye using a highly standardized technique [35]. A dry Dacron polyester-tipped swab (Hardwood Products) was used. Labels with unique identification numbers linked the children’s swab samples and data collection forms. To avoid cross-contamination, the examiner wore a new pair of gloves for each participant. Another fieldworker passed the swab to the examiner, so the examiner only ever touched the stem of the swab.

A total of three swabs were obtained from each patient: (i) one preserved in RNAlater (Ambion Europe) exclusively for RNA extraction, (ii) another preserved in RNAlater for DNA extraction and (iii) a dry swab. All swabs were stored immediately on ice and subsequently at −20 °C. These samples were later processed for RNA and DNA extraction using RNeasy Mini Kit (Qiagen) and QIAamp DNA Mini Kit (Qiagen), respectively, and purified RNA/DNA were collected in a 100 µl volume of elution buffer.

### Detection, quantification and WGS of *Chlamydia trachomatis*

From 345 individuals recruited in the cohort observed every 2 weeks for 28 weeks there were 4830 potential observation points. After withdrawals, absences during collection and sample losses/misidentification we collected 3477 samples. Of these 3386 were successfully tested for chlamydial 16S rRNA as described by our group in a publication evaluating the diagnostic performance of this PCR against CT/NC Amplicor and an *omp*A specific quantitative (q)PCR [32,33, 36]. In total, 3 µl of RNA extracted from the ocular swabs was used as the template in 16S rRNA qPCR. Chlamydial infection is defined as the detection of one or more copies of chlamydial 16S rRNA per reaction in a qPCR assay, which is equivalent to an estimated 16 or more copies per swab.

Quantification of plasmid ORF 2 (pORF2) and *omc*B load was done on the extracted DNA from the swab kept in RNAlater using a droplet digital PCR (ddPCR) technique as described previously [37,38]. In brief, the ddPCR approach enables the conversion of positive and negative droplet counts into an absolute quantification of template numbers within the total PCR volume. This count was subsequently used to estimate copy numbers of the *omc*B gene and pORF2 per microlitre of eluate [37].

Clinical samples with an *omc*B load ≥10 genome equivalents were selected and WGS data were obtained directly from the samples as previously described [3]. Briefly, DNA baits spanning the length of the Ct genome were compiled by SureDesign and synthesized by SureSelectXT (Agilent Technologies). The total DNA extracted from clinical samples was quantified, and carrier human genomic DNA was introduced to achieve a total input of 3 µg for library preparation. DNA was fragmented using a Covaris E210 acoustic focusing unit. Subsequent steps, including end-repair, non-templated addition of 3′-A, adapter ligation, hybridization, enrichment PCR and all post-reaction clean-up processes, were conducted following the SureSelectXT Illumina Paired-End Sequencing Library protocol (v1.4.1, September 2012). DNA was sequenced at University College London/University College London Hospitals Biomedical Research Pathogen Genomics Unit using Illumina paired-end technology (Illumina GAII or HiSeq 2000).

### Trimming, merging and quality control of the sequences

We utilized BBDuk version 38.84 to remove adapters, sequences shorter than 35 bp and those with a Phred quality score below 20 [39]. Additionally, for merging paired reads, we employed BBMerge version 38.84 [39]. Subsequently, trimmed sequences were assessed using FastQC version 0.12.1 for various quality metrics, including ‘per base sequence quality’, ‘per sequence quality scores’, ‘per sequence GC content’, ‘per base N content’, sequence length distribution, and the presence of overrepresented sequences and adaptors [40].

### Sequence assembly and genotyping

Short reads from 43 samples were subjected to a *de novo* assembly using VELVET in conjunction with VelvetOptimiser [41]. The resulting contigs were then mapped to Ct A/HAR13 using Minimap2, specifically employing the long-read spliced alignment data type [42,43]. This facilitated the generation of a consensus sequence, which subsequently served as a foundation for *omp*A genotyping of each sample. The consensus sequence resulting for each sample was further processed for genotyping by extracting the *omp*A gene and comparing its homology against available Ct *omp*A sequences in the blast-n database (https://www.ncbi.nlm.nih.gov/blast/).

### Sequence mapping and annotation

To avoid ambiguity in read mapping, we employed a masking approach for the second copy of the two largest repetitive regions of Ct reference sequences: 16S rRNA_2 and 23S rRNA_2 genes. For mapping short reads, we adopted a genovar-specific strategy, employing Bowtie2 against the masked reference genomes: Ct A/HAR13 and B/Jali20, with a minimum read identity of 90 % and a minimum coverage of 10 [44]. To establish a reliable quality threshold for WGS data, we defined ‘good quality’ as achieving a minimum coverage of 10× across at least 95 % of the Ct reference genome. Chromosomal genes were defined using the annotated genome from Ct strains A/HAR13 and B/Jali20. Annotation of the consensus sequences was done in Geneious using a blast-like algorithm to search for best match annotations with a minimum of 80 % similarity, by aligning the full length of each annotation. In addition, utilizing the blast function in the UniProt database (https://www.uniprot.org/blast), we compared the homology of two hypothetical proteins in Ct SvA sequences with the highest number of detected variations against the annotated genome of Ct strain D/UW-3/Cx. We scanned the whole genomes from Bowtie2 mapping for evidence of antimicrobial resistance using the ABRicate database and staramr version 0.10.0 [45,47].

### Single nucleotide polymorphism/variant calling in individual sequences

For individual samples, the ‘Geneious variations/SNPs caller’ tool was employed to detect single nucleotide polymorphisms (SNPs) among the mapped short reads against the reference genomes: A/HAR13 or B/Jali20. The utilized parameters were as follows: a minimum coverage threshold of 10, a minimum variant frequency threshold of 80 %, a maximum acceptable variant *P*-value of 10^−6^, and a strand-bias *P*-value threshold of 10^−5^, applied only when bias exceeded 65 % (Table 1). Subsequently, SNP accumulation rate per site per year in individual sequences is computed by dividing the number of detected SNPs by 1 044 000 (~Ct genome size) and the number of years, defined by subtracting the sampling year from the year when the reference strain was isolated.

**Table 1.** Specific terminology to this paper

Term	Explanation
SNP	In this context, a single nucleotide polymorphism (SNP) is characterized as a genomic position where the allele frequency is lower than 0.2 when compared to the reference chromosome of *Chlamydia trachomatis* strains A/HAR13 or B/Jali20
SNV	A single nucleotide variant (SNV) was assigned to a population where the reference allele frequency was in the range 10–90 %
Established SNV	An established SNV was assigned to a population at a given site where the reference allele frequency was at least 25 %

### Patterns of single nucleotide variant accumulation within the genes and populations

To analyse the frequency of a single nucleotide variant (SNV) within the SvA compared to the SvB population, we used consensus sequences in FASTQ format to align against their respective reference strains: A/HAR13 or B/Jali20. An SNV was assigned to the SvA or SvB population where the reference allele frequency was in the range 10–90 %, 90 % of the sequences provided coverage and it represented a maximum acceptable variant *P*-value of 10^−6^ (Table 1). Variations with a frequency exceeding 90 % were excluded to minimize those stemming from the use of different reference strains.

To discern the genes accumulating a higher number of variations and those experiencing heightened selective pressure within the SvA population compared to the SvB population, we implemented criteria for calling established SNVs. An established SNV was assigned to a population at a given site if it met the conditions of achieving a variant frequency of at least 25 %, a consensus sequence coverage of at least 90 %, and it represented a maximum acceptable variant *P*-value of 10^−6^ (Table 1). By imposing this 25 % threshold, our aim was to exclude variations that potentially have not become established within the population or result from sequencing errors.

The impact of a variation on a specific codon position dictates whether it leads to an alteration in the amino acid sequence or remains silent [48]. Variations causing amino acid substitutions are classified as non-synonymous, whereas those that do not affect the amino acid sequence are termed synonymous [48,49]. Assessing the proportion of non-synonymous variations is informative when considering the importance of maintaining the coding sequence and indicates whether a gene is subject to evolutionary dynamics of the gene [50]. Therefore, we computed the proportion of non-synonymous to synonymous variations in the Gambian Ct genes and presented them for those experiencing higher selective pressure.

We estimated the type of selection pressure on the Gambian Ct SvA and SvB whole genomes, plasticity zones (PZs) and *omp*A genes by calculating the dN/dS ratio. This ratio infers to the number of non-synonymous substitutions per non-synonymous site (dN) to the number of synonymous substitutions per synonymous site (dS) [49,51]. The dN/dS ratio can indicate neutral evolution (dN/dS=1), positive selection (dN/dS>1), and purifying selection (dN/dS<1). We used the Nei–Gojobori test, with the Jukes–Cantor correction in mega 11 to compute the dN/dS ratio [52,54]. Z-tests of selection were performed with 1000 bootstrap replications to compute the variance of the difference. A positive value signifies an excess of non-synonymous substitutions. Under the null hypothesis of neutral evolution *P*-values of less than 0.05 were considered significant.

### Phylogenetic analysis

For the global phylogenetic analysis of the Ct chromosome, genome sequences from 41 isolates and 29 reference strains were aligned using progressiveMauve (Table S3, available in the online version of this article) [55]. A phylogenetic tree was reconstructed using RAxML (version 8.2.11) and Generalized Time Reversible (GTR) model of evolution with a γ correction for among-site rate variation with four rate categories and 1000 bootstraps [56]. Moreover, plasmids from 41 isolates and 27 reference strains were used to build a phylogenetic tree following the same methodology (Table S3). Here, for the first time, we employed the accurate Ct strain B/Tunis864 genome in drawing the Ct global phylogenetic tree. In the supplementary materials, we have included a concise explanation to address the ongoing confusion pertaining to the labelling of the whole genome sequences of Ct strains B/HAR36 and B/Tunis864 (Data S1 and Table S3).

*omp*A and *trp*AB gene alignments were generated using MAFFT (version v7.490) with a 200 PAM/K=2 scoring matrix (alignment size=1203 and 1957 bp, respectively) [57,58]. For *trp*AB alignment, each of *trp*A and *trp*B was extracted individually and concatenated in Geneious. PhyML was utilized to estimate maximum likelihood phylogenies of aligned sequences with a GTR model of evolution and 1000 bootstraps [59].

### Statistics

Microsoft Excel (version 16.78) and GraphPad Prism (version 10.0.3) were used for designing the graphs. A *P*-value of <0.05 was considered to reflect a statistically significant difference. A non-parametric, two-tailed, Mann–Whitney test was performed to examine any association between Ct infection load and Ct genovar. We used a parametric, unpaired, two-tailed t-test to explore the significance of differences in the distribution of the variations in SvA compared with SvB sequences.

## Results

### Sample collection, *Chlamydia trachomatis* infection and sequencing quality data

The prevalence of ocular infection using 16S rRNA qPCR quantification was 20.9 % (72/345) at recruitment. Over the 6 months of observation 257 individuals tested positive at least once and a total of 1013 positive 16S rRNA qPCR tests were identified. After selecting and using these samples for confirmatory Ct diagnostic tests, and *omp*A, toxin and *tar*P amplicon sequencing tests [30], a subset of 43 representative samples were selected that had sufficient DNA yield, quality and Ct load for WGS. After quality assessments of the whole genome sequences, 41/43 passed defined quality control measures and achieved a minimum coverage of 10× across at least 95 % of the Ct reference genome. The *omp*A genotyping process was carried out on these 41 sequences using NCBI blast-n, which resulted in 11 sequences exhibiting highest similarity to strain A/HAR13 (NC_007429) and 30 sequences to strain B/Jali20 (NC_012686). These 41 samples were derived from 26 participants and originated from four villages (Fig. 1). Among these individuals, 15 sampling time-points (A–O) were documented, and the study samples were ultimately composed of three samples from one patient, two samples from 13 patients and one sample from 12 patients (Table 2). The selected samples originated from participants with a mean age of 8.8 years, consisting of 17 males and nine females, with 22 presenting clinical signs of TF, while four displayed no clinical signs meeting the WHO simplified grading score definition of trachoma (Tables 2 and S1).

**Table 2.** Baseline demographics, trachoma grades and Ct *omp*A type and *omc*B load of study participants

Village	Patient ID	Sex	Age (years)	Trachoma grade	No. of infections	Time between specimens (weeks)	*omp*A genotype	Plasmid genotype	*omc*B copies (μl^–1^)*
Village 1									
	010202D and J	F	5	TF	2	12	B	B	14.8
	010306G and M	F	5	TF	2	12†	B	B	1628
	010404L	F	10	TF	1	–	B	B	37.3
	010702D and K	F	7	TF	2	14	B	B	56.4
	010703D and J	F	4	TF	2	12	B	B	1112.6
	010705I	M	10	Normal	1	–	B	B	68.8
	011402D and I	F	10	TF	2	10	B	B	1960.7
	011501C and J	F	5	TF	2	14	B	B	1551.2
	011502C and J	F	4	TF	2	14	B	B	193.1
Village 4									
	040104A	M	6	TF	1	–	A	A	249.3
	040119A and G and L	M	13	TF	3	12 and 8	A	A	27.1
Village 6									
	060121A and K	M	8	TF and normal	8	20	A	A	358.6
	060123G	M	7	TF	7	–	A	A	216
	060124L	F	8	TF	8	–	A	A	24.7
Village 9									
	090109J and N	M	15	TF and normal	2	8	B	B	408
	090120 N	M	9	Normal	1	–	B	B	69.3
	090132 N	M	13	TF	1	–	A	A	29.5
	090134J and L	M	9	TF	2	4	B	B	114.3
	090139L	M	9	TF	1	–	B	B	34.5
	090142 N	M	13	TF	1	–	B	A	27
	090154J and L	M	11	Normal	1	–	B	B	69
	090155 N	M	9	TF	2	4	B	B	297.3
	090167 N	M	9	Normal	1	–	B	B	28.8
	090168L and N	M	10	TF and Normal	2	4	B	B	278.2
	090172J and M	M	11	TF	2	6	A	A	933.6
	090195 N	M	8	TF	1	–	B	B	37.5

*To calculate the average *omc*B load for samples with multiple infection time-points, we aggregated data from all time-points.

†Among those from which we obtained WGS data in more than one sampling time-point, only 010306 remained constantly PCR-positive between two sampling time-points (12 weeks).

On average, each sample generated 3 540 540 raw reads, with 639 183 merged paired-reads successfully mapped to the Ct reference genome. Across these 41 samples, the average coverage of the Ct reference genome was 113 (merged paired-reads), with a corresponding mean confidence score of 38 (Table S2).

### *omp*A diversity and *omc*B copy number

Maximum blast-n homology against the Ct *omp*A assigned 11 samples to Ct SvA, strain A/HAR13, and 30 samples to Ct SvB, strain B/Jali20. Serovar distribution across villages was as follows: 16 samples were classified as SvB in village 1, four samples as SvA in village 4, four samples as SvA in village 6, and three samples as SvA and 15 samples as SvB in village 9 (Table 2, Fig. 1). The average *omc*B gene copy number for SvB variants (420.4 copies μl^−1^) was higher than that for SvA variants (270.5 copies μl^−1^), but the difference was not significant (*P*=0.6597).

**Fig. 2.**
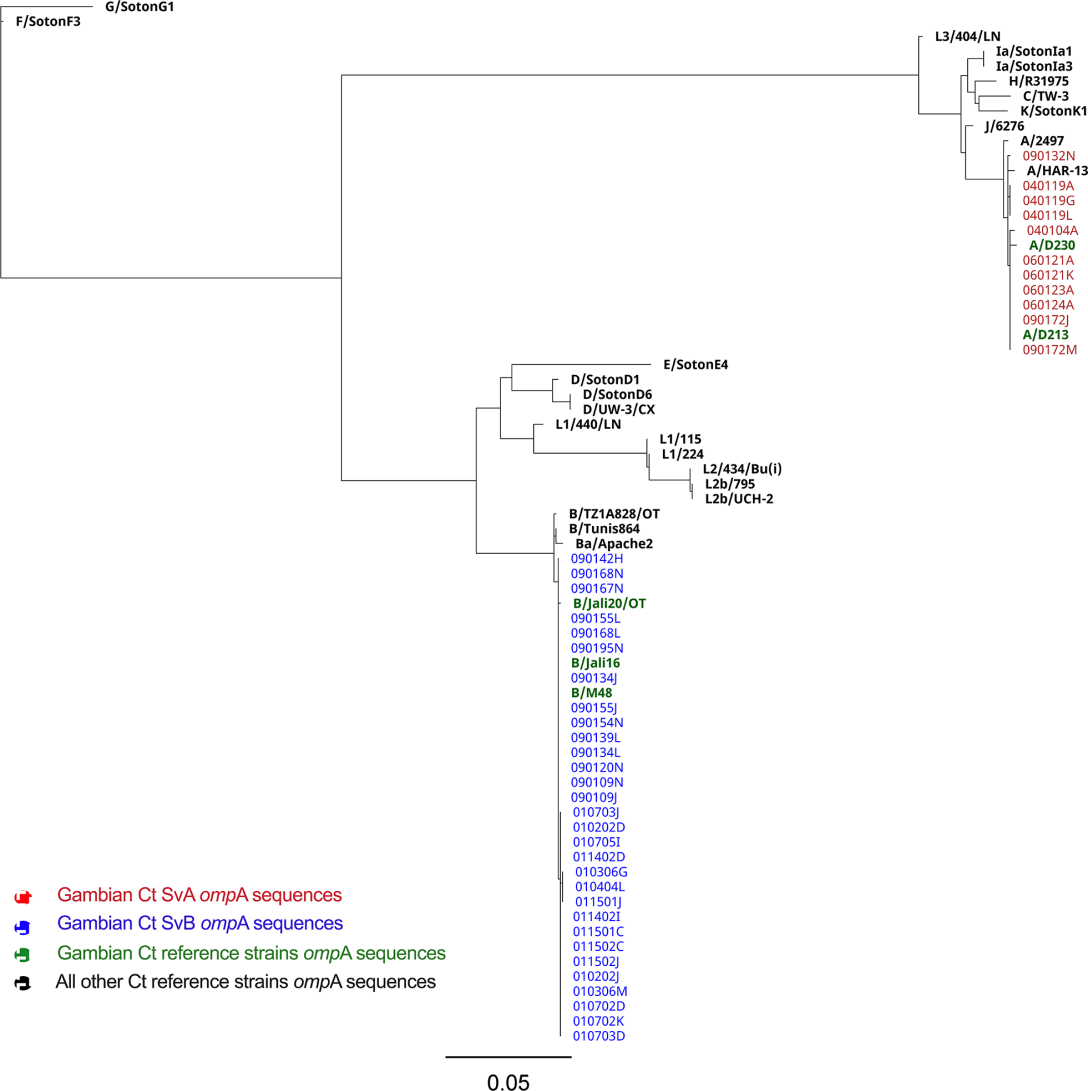
Maximum likelihood phylogenetic reconstruction of ocular *Chlamydia trachomatis omp*A sequences from The Gambia. The phylogenetic analysis encompasses 41 *C*. *trachomatis* (Ct)-positive samples collected in The Gambia and 29 Ct reference strains. The initial two digits of each sample identifier signify the respective recruitment village. The Gambian samples testing positive for Ct serovar A are denoted in red, while those for serovar B are indicated in blue. All reference strains are highlighted in bold, with strains originating from The Gambia, A/D213, A/D230, B/Jali16, B/Jali20 and B/M48, represented in green. Bar, evolutionary distance of 0.05.

In line with blast-n results, phylogenetic analysis of *omp*A assigned the Gambian sequences to two distinct clusters (Fig. 2) where SvA sequences grouped closely with Ct strain A/HAR13 (isolation year: 1958) [60], and two SvA Gambian reference strains:, A/D213 (isolation year: 2001) [61] and A/D230 (isolation year: 2001) [18]. Moreover, all three SvB reference strains from the Gambia, B/Jali16 (isolation year: 1985) [60], B/Jali20 (isolation year: 1985) [62] and B/M48 (isolation year: 2007) [18], grouped together with SvB sequences from village 9. Among sequences classified as SvB, a unique substitution was observed in sequences from village 1 at position 893 (C>T=A>V) of *omp*A that differentiated these sequences from SvB sequences from village 9 and the Gambian SvB strains deposited previously in the ENA: B/Jali16, B/Jali20 and B/M48 [18,60, 62]. Moreover, two substitutions at positions 186 (G>A=M>I) and 268 (G>A=A>T) of *omp*A were specific to the SvB sequences that originated from The Gambia including those from our study.

### Global phylogeny of the Gambian *Chlamydia trachomatis* genomes and plasmids

**Fig. 3.**
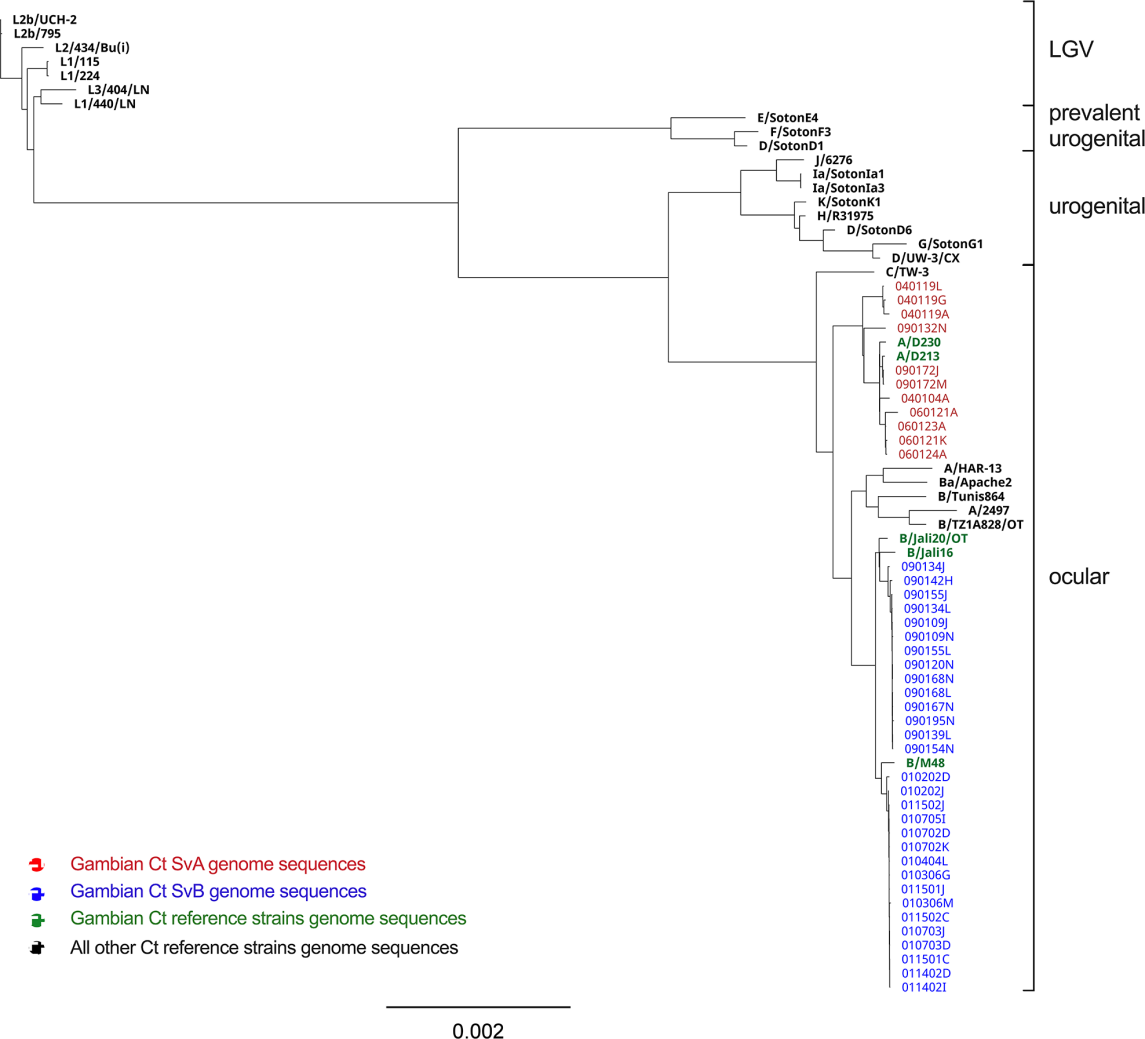
Global phylogeny of *Chlamydia trachomatis* chromosomal sequences from the Gambia. The four major *C. trachomatis* (Ct) lineages are listed on the right. Chromosomal sequences were aligned using progressiveMauve, and a phylogenetic tree was reconstructed employing RAxML, incorporating the Generalized Time Reversible (GTR) model of evolution with a γ correction for among-site rate variation, employing four rate categories, and subjected to 1000 bootstrap resampling iterations. Whole genome sequences of the Gambian samples testing positive for Ct serovar A are denoted in red, while those for serovar B are indicated in blue. All reference strains are highlighted in bold, with strains originating from The Gambia, A/D213, A/D230, B/Jali16, B/Jali20 and B/M48, represented in green. Bar, evolutionary distance of 0.002.

We studied the phylogenetic distribution of SvA and SvB genomes derived from our study and a selection of chlamydial genomes that corresponded to the four major Ct clades found globally, including LGV, UGT, prevalent UGT and ocular clades [18,61]. To better resolve the phylogeny of the Gambian Ct genomes, we introduced three less familiar genome sequences from previous Gambian studies: A/D213 [35], A/D230 [18] and B/M48 [18], alongside the known SvB reference strains B/Jali16 [34] and B/Jali20 [36]. All Gambian genomes clustered within the ocular clade, forming two distinct subclades of SvA and SvB (Fig. 3). This suggests that the Gambian genomes were derived from at least two separate ancestral sources. Remarkably, the SvA subclade may represent a locally endemic clone, distinct from the more common SvA reference strains including A/HAR13 and A/2497 (Fig. 3).

Within the Gambian SvA subclade, we observed two distinct groups. The first comprises three genomes isolated from a single individual in village 4 (Fig. 3). The second group includes one genome from village 4, along with genomes from village 6 and village 9 (Fig. 3). These genomes share a common ancestor with reference strains A/D213 and A/D230. Sequences from village 6 form a separate subgroup within the second group (Fig. 3). Surveying the SvB subclade, we noted that sequences from village 1 and village 9 divide into two distinct groups (Fig. 3). SvB genomes from village 9 cluster together and share a common ancestor with strain B/Jali20, and SvB genomes from village 1 share a common ancestor with strains B/M48 and B/Jali16 (Fig. 3).

**Fig. 4.**
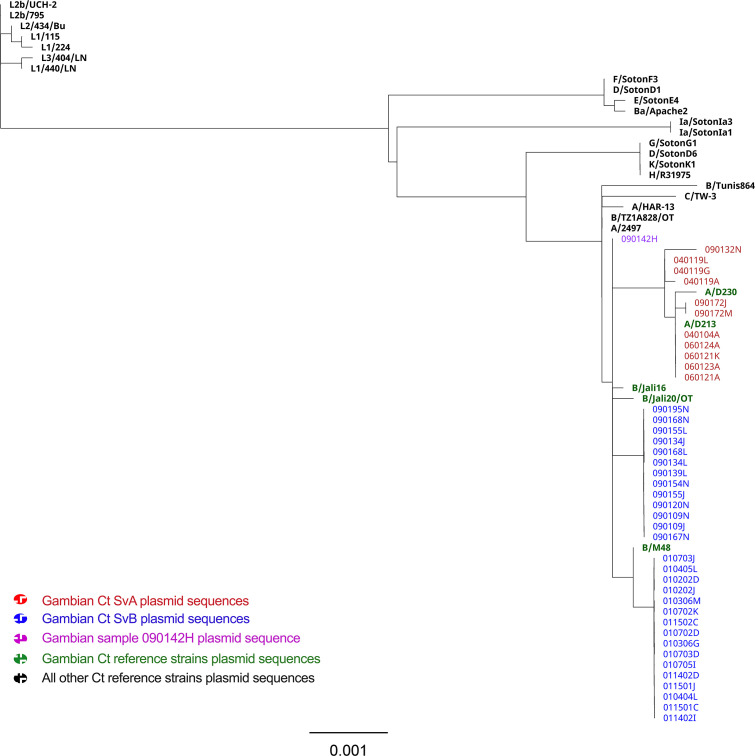
Global phylogeny of *Chlamydia trachomatis* plasmid sequences from The Gambia. In total, 41 Gambian and 27 *C*. *trachomatis* (Ct) reference strain plasmid sequences were aligned using progressiveMauve, and a phylogenetic tree was reconstructed employing RAxML, incorporating the Generalized Time Reversible (GTR) model of evolution with a γ correction for among-site rate variation, employing four rate categories, and subjected to 1000 bootstrap resampling iterations. Plasmid sequences from the Gambian samples testing positive for Ct serovar A are denoted in red, while those for serovar B are indicated in blue. The plasmid sequence of sample 090142H formed a separate branch from the rest of the Gambian sequences and is marked in violet. All reference strains are highlighted in bold, with strains originating from The Gambia, A/D213, A/D230, B/Jali16, B/Jali20 and B/M48, represented in green. Bar, evolutionary distance of 0.001.

Like the Ct chromosome phylogeny, the global phylogeny of Ct plasmid reveals four distinct clusters: ‘LGV’, ‘UGT’, ‘prevalent UGT’ and ‘ocular’ clades, with the exception of strain Ba/Apache2 that grouped within the ‘prevalent UGT’ cluster (Fig. 4) [50]. Gambian plasmids formed one distinct subclade within the ocular clade from all other ocular reference strains. We identified six branches within the Gambian subclade including (i) sequences from Ct SvA isolates; (ii) sequences from SvB isolates from village 9; (iii) sequences from SvB isolates from village 1 that share an ancestor with the reference strain B/M48; (iv) reference strain B/Jali16; (v) reference strain B/Jali20; and (vi) one plasmid from village 9 (090142H) that did not group with any other plasmids from village 9 and formed a separate branch (Fig. 4). Intriguingly, despite being classified as SvB based on *omp*A and WGS data, examining the blast-n homology of 090142H plasmid, it demonstrated the highest homology to a plasmid of Ct strain A/2497. Looking at the Gambian Ct SvA plasmid sequences, three plasmids from one participant in village 4 (040119A, G, L) grouped together with one plasmid from village 9, while all other plasmids formed a separate subgroup sharing common ancestors with the reference strains A/D213 and A/D230 (Fig. 4).

Among Ct SvA plasmid sequences we found seven SNVs with a frequency of at least 25 % from which three were non-synonymous. Three (43 %) out of seven variations accumulated in CDS3 (replicative DNA helicase), while four other variations accumulated in CDS1 (14.3 %), CDS2 (14.3 %), CDS7 (14.3 %) and CDS8 (14.3 %). Two out of three non-synonymous variations accumulated in CDS3 and one in CDS1 (pgp7). In comparison, we found eight established SNVs among Ct SvB plasmid sequences from which two were non-synonymous. The distribution of the established SNVs on the plasmid of Ct SvB isolates was as follows: two (25 %) in CDS1, two (25 %) in CDS2, one (12.5) in CDS3, two (25 %) in CDS4 and one (12.5 %) in CDS8. Non-synonymous variations were found in CDS1 and CDS4.

### Single nucleotide polymorphism accumulation in individual chromosome sequences

We identified fixed mutations in individual samples as those where 80 % of the sequencing reads with a minimum coverage of 10 disagreed with the reference base. In the case of SvA sequences, the range of SNPs was between 1117 and 1175, while for SvB sequences, it ranged from 144 to 295 (*P*<0.0001). On average, among SvA sequences from villages 4, 6 and 9, there were 1156, 1132 and 1169 SNPs, respectively. Conversely, among SvB sequences from villages 1 and 9, the average number of SNPs detected was 285 and 238, respectively (*P*<0.0001). Considering the respective years in which the samples were collected, Ct strain A/HAR13 in 1958 and Ct strain B/Jali20 in 1985, the SNP accumulation rates for our Gambian SvA and SvB variants were calculated as ~2.5×10^−5^ and ~1.4×10^−5^ per site per year, respectively (*P*<0.0001) [60,62].

### Variation frequency among the Gambian sequences

**Fig. 5.**
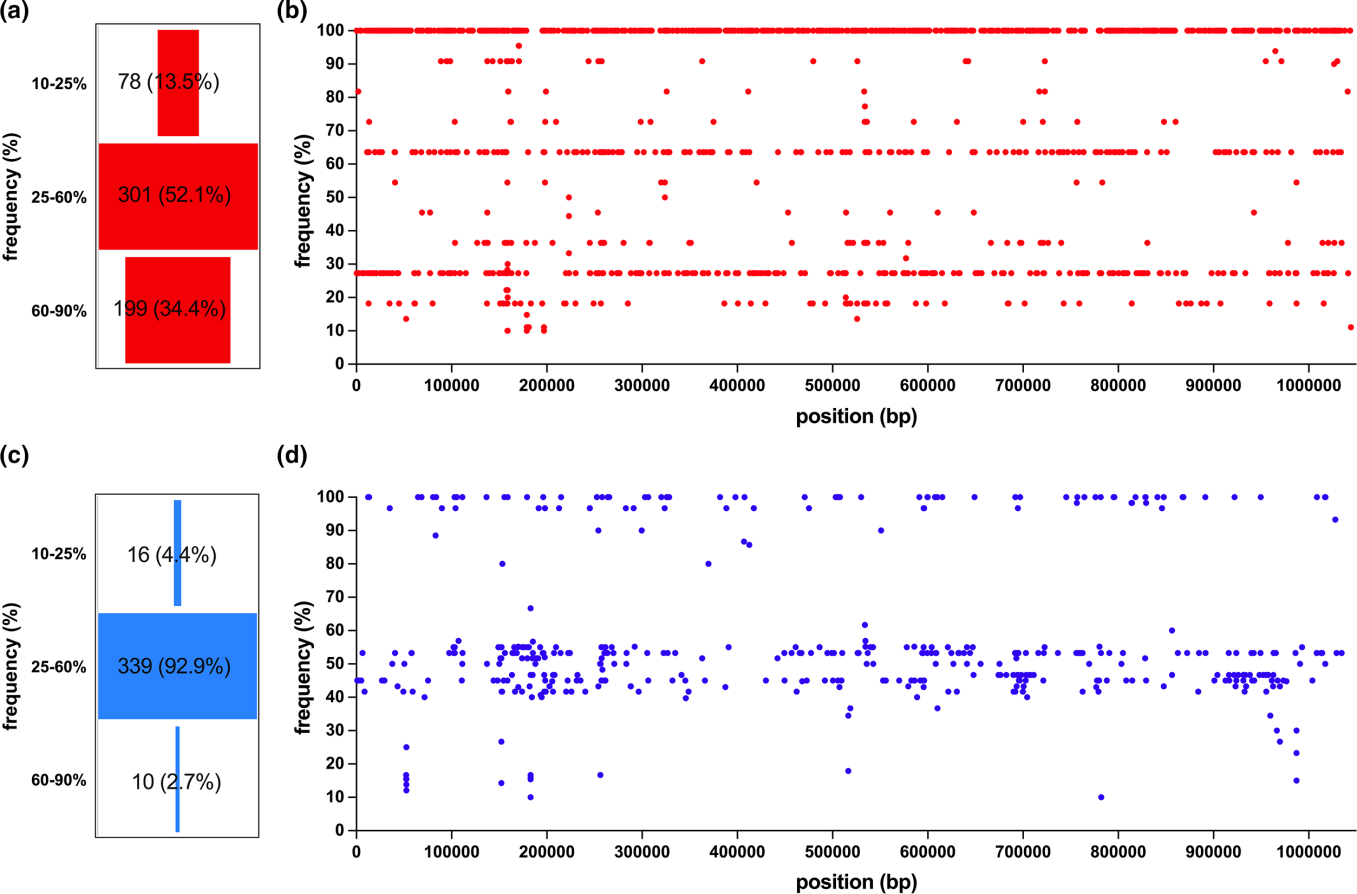
Variation frequency analysis in the Gambian *Chlamydia trachomatis* variants. In (**a**) and (**b**), the red funnel and dot plot graphs display the frequency of single nucleotide variants (SNVs) accumulated in the Gambian serovar A strains in relation to the reference strain A/HAR13. Conversely, in (**c**) and (**d**), the blue funnel and dot plot graphs illustrate the frequency of SNVs accumulated in the Gambian serovar B strains in comparison to the reference strain B/Jali20. The funnel exclusively incorporates SNVs with a frequency ranging from 10 to 90%.

To assess the frequency of the variations within the sequences classified as SvA and SvB, a variation was assigned where the reference allele frequency was in the range 10–90 %. Based on the variation frequency, we categorized them into three groups: (i) those with a frequency of 10–25 % were considered not established at the village level; (ii) SNVs with a frequency of 25–60 % were deemed established in one village; and (iii) SNVs with a frequency of 60–90 % were regarded as established in at least two villages. For both SvA and SvB populations the majority of the SNVs (52.1 and 92.9 %, respectively) were established in one village (Fig. 5). For the SvA population while 34.3% of the SNVs were in common among the residents of at least two villages (Fig. 5a, b), only 2.7% of the SNVs in the SvB population could become established in both village 1 and 9 (Fig. 5c, d). Furthermore, we noted a higher number of unestablished SNVs at a village level in the SvA (13.5 %) population compared to the SvB (4.4 %) population (Fig. 5).

### Gambian serovar A strains appear under higher purifying selection than serovar B strains

For both Gambian SvA and SvB whole genome sequences strict-neutrality (dN=dS) was rejected in favour of the alternative hypothesis (dN<dS; *P*=0.00031 and 0.01), with evidence indicating a higher purifying effect on SvA sequences (dN/dS=0.61) compared with SvB sequences (dN/dS=0.7). Analysing variations with a frequency of at least 25 % yielded a total of 1371 established SNVs among SvA sequences and 438 established SNVs among SvB sequences. Among the SvA sequences, 740 established SNVs (62.8 %) were categorized as non-synonymous. In contrast, within the SvB sequences, 241 (66.4 %) were designated as non-synonymous.

### *Chlamydia trachomatis* genes under highest selective pressure

While the established SNV rates on the chromosomes of SvA and SvB variants are ~13.2 and ~4.2/10 kb, respectively, it is noteworthy that the PZ, spanning from *acc*B to *trp*A, exhibits a heightened established SNV rate for both SvA (~20.2 mutations/10 kb) and SvB (~10.4 mutations/10 kb). Similar to SvA and SvB whole genomes, the null hypothesis of strict-neutrality (dN=dS) for SvA and SvB PZ was rejected in favour of the alternative hypothesis (dN<dS; *P*=0.02 and 0.0036, respectively). However, in contrast to whole genomes, SvB PZ (dN/dS=0.34) appears to experience higher levels of purifying selection pressure than SvA PZ (dN/dS=0.53).

*omp*A of SvA strains was found to be under positive selection (dN>dS; *P*=0.02), but the dN–dS test statistic was not significant for the *omp*A gene of SvB sequences (*P*>0.05), suggesting that SvB *omp*A did not significantly depart from neutrality [50]. We recorded five established SNVs (four non-synonymous and one synonymous) in *omp*A of SvA sequences and two established SNVs (both non-synonymous) in *omp*A of SvB sequences. A variation at position 268 (G>A=A>T) of the Gambian SvB *omp*A was located on variable domain 1.

**Fig. 6.**
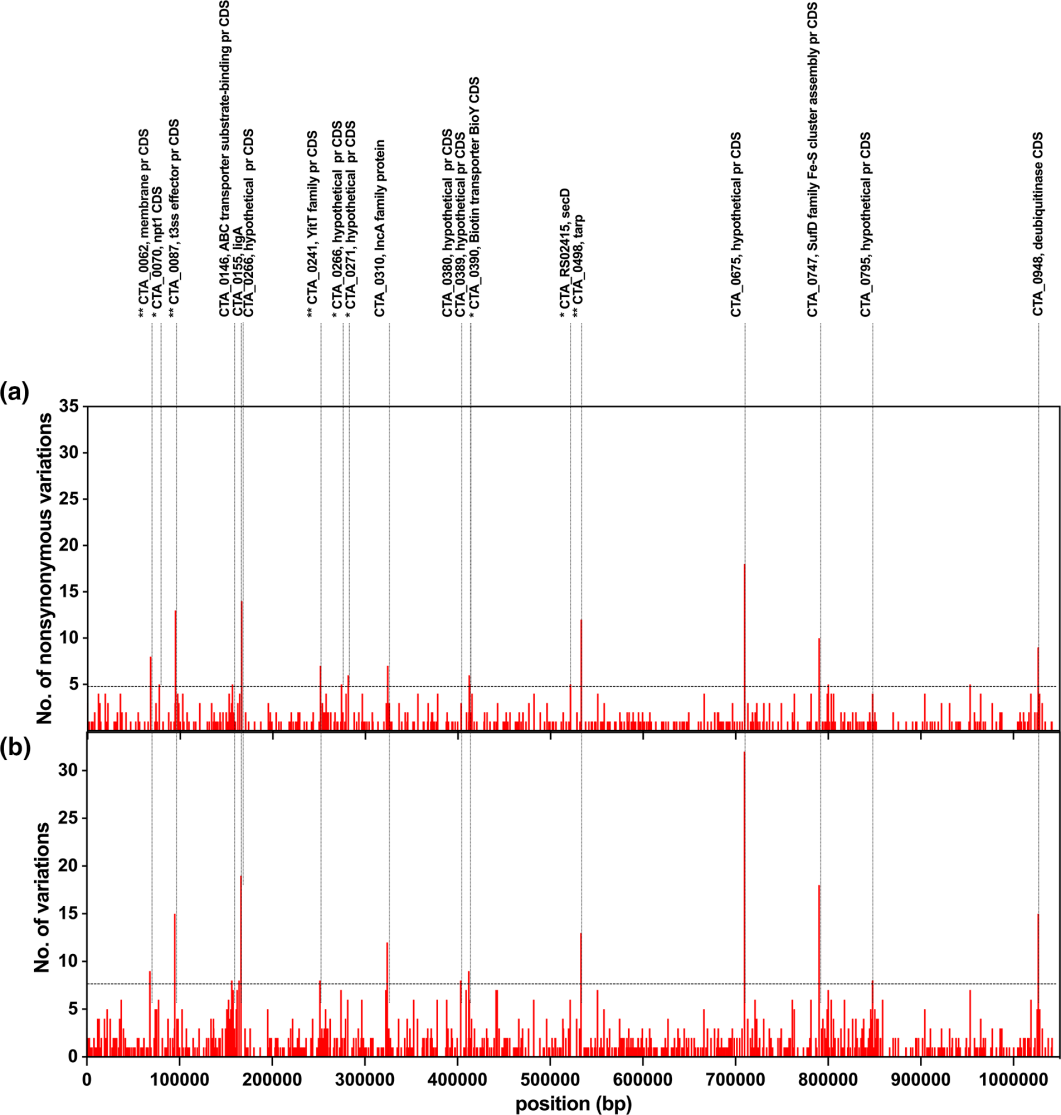
Gene-level variation accumulation profiles in *Chlamydia trachomatis* SvA variants. (**a**) The total number of variant sites (upper bars) detected for each *C. trachomatis* gene, and (**b**) the number of non-synonymous substitution sites (lower bars) exceeding 25% frequency among the Gambian SvA sequences. The chromosomal genes are ordered according to genome annotation of the A/HAR13 strain (NC_007429). Genes showing at least five non-synonymous substitution sites (and no synonymous variant sites) or a proportion of non-synonymous to synonymous variations of at least 5 : 1 (Top 1 %) are labelled above the graph. Genes showing at least eight total established SNV sites (Top 1 %) are labelled above the graph. A single asterisk (*) labels genes with the highest proportion of non-synonymous to synonymous variations (Top 1 %). A double asterisk (**) labels genes that were in common between those with the highest proportion of non-synonymous to synonymous variations, and highest number of established SNV sites (Top 1 %).

Examining the genes that have accumulated the highest number of established SNVs and proportion of non-synonymous to synonymous variations in Ct SvA sequences (Tables S4 and S5, Fig. 6a, b), we can categorize these genes based on their functional significance as follows: (i) genes involved in energy/nutrition/protein transport and trafficking pathways including CTA_0070/*npt*1, CTA_0087 (T3SS), CTA_0146, CTA_0156, CTA_0241 (YitT), CTA_0251, CTA_0390/*bio*Y, CTA_RS02415/*sec*D and CTA_0747/*suf*D; (ii) genes pivotal for Ct virulence, such as CTA_0310/*inc*A, CTA_0498/*tar*P, CTA_0675 and CTA_0948 (deubiquitinase, DUB); (iii) genes important for Ct membrane structure, exemplified by CTA_0062, CTA_0271 and CTA_0389; and (iv) those important for Ct DNA replication and repair, represented by CTA_0155/*lig*A [60,63,74]. Of note, the highest number of SNVs were detected in two Ct SvA hypothetical protein genes; CTA_0156 starting at position 166384 and CTA_0675 starting at position 709788 with 19 and 32 SNVs. blast function in the UniProt database indicated that CTA_0156 shares 97.9% homology with CT_147 and CTA_0675 shares 96.3% homology with CT_622 in Ct strain D/UW-3/Cx. A study by Belland *et al*. [75] suggested that CT_147 is an immediate early-gene (1 h post-infection) and a homologue of the human early endosomal antigen-1 that is localized to the chlamydial phagosome, establishing a parasitophorous vacuole in a non-fusogenic pathway. However, a later study by Cortina *et al*. [76] contradicted these results, suggesting that CT_147 impacts the elementary body (EB) to reticulate body (RB) transition during the early stages of chlamydia development. Studies by Hamaoui *et al.* [72] and Cosse *et al.* [77] introduced CT_622 as an early gene (2 h post-infection) and a multifunctional effector in Ct. These studies revealed that genetic disruption of CT_622 expression resulted in a strong bacterial growth defect, which was due to deficiencies in proliferation and in the generation of infectious bacteria [72,77].

**Fig. 7.**
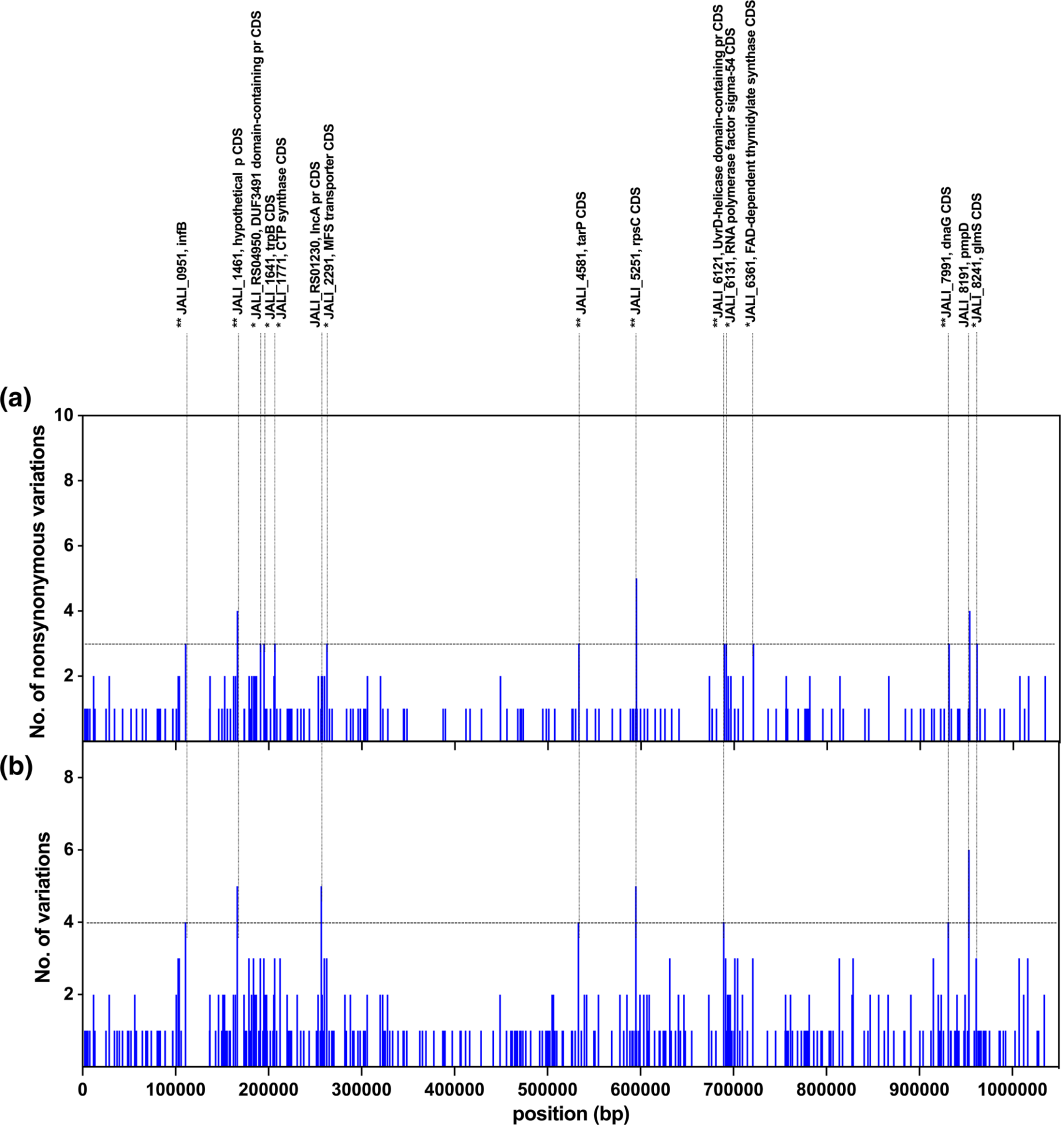
Gene-level variation accumulation profiles in *Chlamydia trachomatis* SvB variants. (**a**) The total number of variant sites (upper bars) detected for each *C. trachomatis* gene, and (**b**) the number of non-synonymous substitution sites (lower bars) exceeding 25% frequency among the Gambian SvB sequences. Chromosomal genes are ordered according to genome annotation of the B/Jali20 strain (NC_012686). (**a**) Genes showing at least three non-synonymous substitution sites (and no synonymous variant sites) or a proportion of non-synonymous to synonymous variation of at least 3 : 1 (Top 1 %) are labelled above the graph. (**b**) Genes showing at least four total established single nucleotide variant (SNV) sites (Top 1 %) are labelled above the graph. A single asterisk (*) labels genes with the highest proportion of non-synonymous to synonymous variation (Top 1 %). A double asterisk (**) labels genes that were in common between those with the highest proportion of non-synonymous to synonymous variation, and highest number of SNV sites (Top 1 %).

SvB genes with the highest established SNV counts and proportion of non-synonymous to synonymous variations are distributed among four categories (Tables S4 and S5, Fig. 7a, b). The largest group encompasses (i) genes involved in DNA replication, repair and RNA synthesis, notably JALI_0951/*inf*B, JALI_5251/*rps*C, JALI_6121 (UvrD helicase) and JALI_6131, JALI_7991/*dna*G [78,82]. The other groups include (ii) genes relevant to amino acid synthesis and metabolic processes, such as JALI_1641/*trp*B, JALI_1771, JALI_6361 and JALI_8241/*glm*S; (iii) genes associated with Ct virulence, including JALI_RS01230/*inc*A, JALI_4581/*tar*P and JALI_8191/*pmp*D; and (iv) genes participating in transport and trafficking pathways, exemplified by JALI_2291 [66,69, 83,88].

### Tryptophan operon analyses revealed a truncation in TrpB

Maximum likelihood phylogenetic analysis of the *trp*AB genes resulted in the formation of a separate clade specific to the ocular strains (Fig. S1). Within this ocular clade, the Gambian *trp*AB sequences can be further divided into three distinct subclades: (i) SvB sequences only from village 1, along with the reference strain B/M48; (ii) seven SvA sequences derived from villages 4, 6 and 9, in addition to the reference strains A/D213 and A/D230; and (iii) SvB sequences from village 9, which cluster together with four SvA sequences from villages 4 and 9 (Fig. S1).

Examination of the *trp*B gene revealed the presence of an insertion spanning two nucleotides at positions 1315 and 1316 (G and A, respectively). This insertion event resulted in a frameshift, leading to the early termination of the *trp*B CDS. Notably, this mutation is conserved across all SvB sequences originating from village 1 and is also present in the reference strain B/M48 (Fig. S2).

### Short-term mutation accumulation trends in *Chlamydia trachomatis* SvA and SvB

We obtained WGS data from three individuals who tested repeatedly positive for SvA in villages 4, 6 and 9, as well as 11 individuals who tested repeatedly positive for SvB in villages 1 and 9. On average, the time interval between the first and second identified infections used for WGS for SvA sequences was ~12.7 weeks, while for SvB sequences, it was ~9.8 weeks. A comparison between the first and second infection within each participant revealed a total of 171 SNP events (33 among SvA sequences and 138 among SvB sequences), resulting in an average of 11 SNPs per SvA genome and 11.5 SNPs per SvB genome. Notably, we identified 35 SNPs within Ct PZ. This represents an elevated SNP rate, averaging at 5.8 SNPs/10 kb, which is nearly four times higher than the average observed rate (1.6 SNPs/10 kb) across the entire Ct genome.

**Fig. 8.**
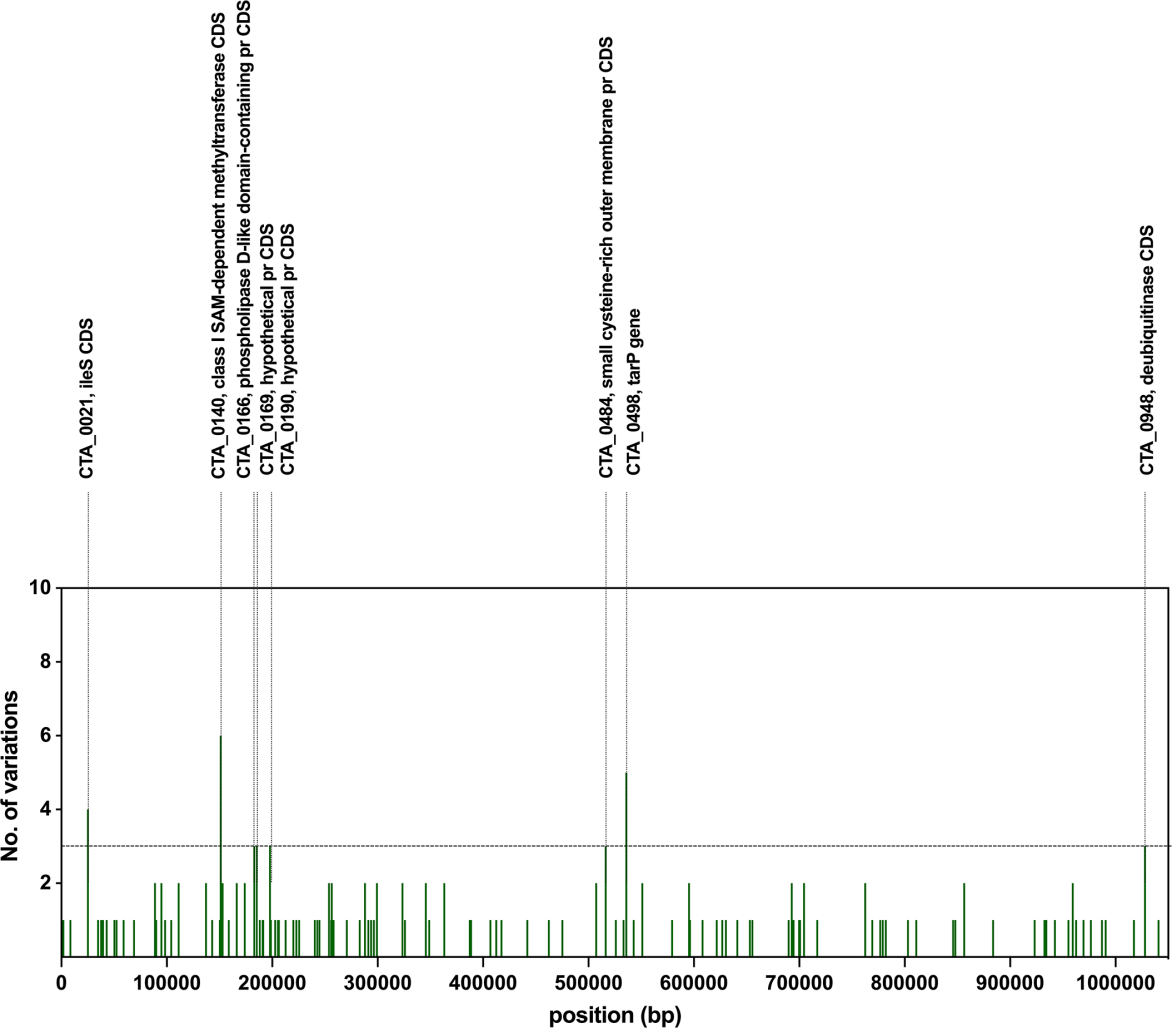
Short-term mutation accumulation profile in *Chlamydia trachomatis* SvA and SvB sequences. Each column represents the cumulative mutation count detected between first and second infections within each *C. trachomatis* gene across three participants who tested positive for serovar A and 11 participants who tested positive for serovar B. The analysis average timespan was 11.25 weeks. Chromosomal genes are ordered according to genome annotation of the A/HAR13 strain (NC_007429).

Table S6 and Fig. 8 represent the top 1 % of Ct genes with the highest number of mutation events detected between first and second infections. Sequence comparisons between first and second infections revealed four categories of the genes that have accumulated the majority of the SNPs including (i) genes associated with Ct virulence such as CTA_0498/*tar*P, CTA_0166 (phospholipase D-like protein PDL), and CTA_0948 (DUB); (ii) CTA_0021/*ile*S associated with amino acid metabolic process; (iii) CTA_0484/*omc*A associated with Ct extracellular matrix; and (iv) CTA_0140 that is involved in transport and trafficking pathways (Fig. 8 and Table S6) [66,73, 75, 89,91].

## Discussion

We conducted WGS on Ct isolates obtained from 11 SvA and 30 SvB variants from The Gambia. Of note, samples positive for Ct SvA originated from three villages [village 4 (Jokadu), 6 (Lower Niumi) and 9 (Central Baddibu)] in the North Bank Region of the river and samples positive for Ct SvB originated from two villages, one in the North Bank Region (village 9) and one in the West Coast Region [village 1 (Kombo South)] (Fig. 1). We first looked at the global phylogeny of the Gambian sequences and suggested that the Gambian SvA sequences may represent a locally endemic clone distinct from the known Ct SvA reference strains A/HAR13 and A/2497. Then, by comparing the SNP accumulation rate among the sequences we showed a higher rate of SNP accumulation in the Gambian SvA sequences than in SvB sequences. Finally, we examined the variation accumulation and frequency in SvA genes and sequences relative to SvB genes and sequences, respectively, which suggested that in SvA isolates genes responsible for host cell modulation and intracellular survival mechanisms are under the highest evolutionary pressure, whereas in SvB variants this pressure was mainly on genes essential for DNA replication/repair mechanisms and protein synthesis. We further propose that distinctive patterns in variation frequency between SvA and SvB sequences may, in part, be attributed to geospatial differences in the distribution of the Gambian sequences.

Through phylogenetic analysis of Ct *omp*A sequences, we showed that diversification in the Gambian SvA *omp*A sequences is not driven by the location of the villages, whereas for SvB sequences there is a distinction between sequences from village 1 and 9. Of note, both SvA and SvB *omp*A sequences grouped closely with Ct A/HAR13 and B/Jali20, sequences collected ~50 and ~20 years prior to this study [60,62]. Previously, studies on Ct UGT strains showed that the expansion of genotype E, currently the most prevalent UGT genotype, may be due to increased fitness at or around *omp*A, preventing recombination being fixed in this region, being simply a stochastic increase or being a combination of the two [18].

Of note, differences in the frequency of the SNVs among SvA and SvB sequences support the global phylogeny of the Gambian Ct chromosomes. There is a higher influx of the unestablished SNVs (ranging from 10 o 25 %) among the SvA population (13.5 %) than among the SvB population (4.4 %). This may explain the higher phylogenetic diversity observed within the Gambian SvA sequences in contrast to the SvB sequences. Our findings on SvB sequences support a prior observation by Alkhidir *et al.* [15] suggesting a similar phylogenetic relatedness for two SvB Gambian isolates collected ~20 years apart: B/Jali20 and B/M48, indicating slow and geographically related diversification. The majority of the SNVs for both SvA (52.1 %) and SvB (92.9 %) sequences were established on a village level (ranging from 25 to 60 %). This prevalence of village-level SNVs among SvB sequences probably contributes to the formation of distinct clusters within SvB sequences, corresponding to their respective villages of origin. This supports previous findings in trachoma endemic populations that suggest geographical clustering of ocular Ct strains [13,18, 92]. Moreover, while 34.4% of the SNVs could reach a frequency of 60–90 % and become established among the majority of the SvA population, potentially accounting for the emergence of a distinctive Gambian SvA subclade within the traditional ocular Ct clade, only 2.7% of the SNVs among the SvB population could reach to a frequency of 60–90 %.

In line with previous studies that demonstrated concordance of chromosome and plasmid phylogeny [11,61, 93, 94], the phylogenetic position of the Gambian plasmids, with the exception of one sample, is consistent with the whole genome phylogeny. blast-n analysis of the sample 090142H plasmid showed the highest homology to Ct SvA strain A/2497, while *omp*A and chromosome phylogeny classified this sample as SvB. Previous studies on UGT strains presented rare evidence of horizontal plasmid transfer events, recombination events and plasmid swapping [18,93, 95]. While our data reveal a strong association between the chromosomal genotype and plasmid that suggests their co-evolution, there remains a possibility of recombination or a swapping event for the plasmid of 090142H.

Previous data for Ct LGV strains and *Chlamydia psittaci* estimated substitution rates of 2.1×10^−7^ and 1.7×10^−4^ SNPs per site per year [18,96, 97]. Our results estimated 2.5×10^−5^ and 1.4×10^−5^ SNPs per site per year for the Gambian SvA and SvB variants, respectively [60,62]. This suggests an almost two times higher SNP accumulation rate among the Gambian SvA sequences compared with SvB sequences, and approximately 100–200 times higher substitution rate in the Gambian ocular strains than that reported for Ct LGV [18,96]. This higher rate can be in part due to the differences in Ct serovars and might also be driven by acquisition and recombination events between Ct strains in the Gambian populations [18,96]. The dN/dS ratio is a measure of selective pressure [98], where ratios of <1 indicate purifying selection [49,99]. We found a higher purifying selection on the Gambian SvA sequences than on the Gambian SvB sequences. Moreover, the ratio of dN/dS in the Gambian SvB sequences was close to 1, indicating a high stabilizing selection on the SvB sequences. In line with our results, a study by Joseph *et al.* [11] reported higher levels of purifying selection on Ct SvA strain A/HAR13 compared to Ct SvB strain B/Jali20.

In agreement with previous data [28], we observed a higher Ct load for SvB compared with SvA variants. Previously, Kari *et al.* [100] provided evidence demonstrating a direct relationship between polymorphisms in specific Ct genes and virulence properties of trachoma strains. In SvA sequences, genes linked to host cell modulation (e.g. ‘DUB’), host–pathogen interactions (e.g. ‘T3SS effector protein’), and intracellular survival and nutrient acquisition (e.g. ‘*npt*1’), and in SvB sequences, genes related to RNA translation (e.g. ‘*inf*B’), DNA replication (e.g. ‘*dna*G’) and amino acid synthesis (e.g. ‘*trp*B’) accumulated the highest number of established SNVs and/or proportion of non-synonymous to synonymous variations [63,64, 73, 74]. These genes in the Gambian SvB strains potentially lead to increased replicative fitness of Ct within the host cell [78,82, 88]. Remarkably, the most substantial count of SNVs was identified in two hypothetical protein genes within Ct SvA sequences: CtA_156 and CtA_675. Previous research has highlighted the significance of these genes in the intracellular survival and growth of Ct in genital and LGV strains [72,75,77]. Further investigations on these genes may provide valuable insights into their function and reasons behind their heightened evolutionary pressure among the Gambian ocular Ct SvA strains. A study conducted by Sigalova *et al.* [101] classified chlamydial genes into three functional groups based on annotated Clusters of Orthologous Genes. Combining this classification with our observations indicates that a larger proportion of ‘core genes’ among SvB than SvA variants experience greater evolutionary pressure. Conversely, a higher number of ‘periphery genes’ among SvA compared to SvB variants are subject to increased selective pressure.

Ct urogenital strains have been classified as tryptophan (Trp) prototrophs, using indole produced by other microbiota to synthesize Trp within a closed-conformation tetramer consisting of two α (TrpA)- and two β (TrpB)-subunits [102,105]. On the other hand, ocular strains are auxotrophs, as they carry mutations in TrpA, necessitating them to depend on the host’s available Trp pools for their survival [104,105]. While our results are in line with previous studies and indicate a frameshift leading to truncation in Ct TrpA in the Gambian samples [106,107], we reported an insertion in the *trp*B gene of SvB sequences from village 1 that causes a frameshift, and therefore the truncation of TrpB. This observation is supported by a previous study on the Gambian Ct B/M48, a strain that was isolated almost 4 years after our samples [18]. Gene inactivation and forming pseudogenes through truncation is a common mode of evolution in bacteria [108,110]. Pseudogenization is prevalent in intracellular bacteria, resulting from reductive evolution following ‘use-or-lose’ dynamics to allow purging of traits since the genes being inactivated are of no use in the organism [109,110].

Our study is subject to several limitations that warrant consideration. Limited participants with repeated SvA infections challenge understanding of short-term SNP accumulation patterns in SvA variants. A larger sample size from wider geographical regions in The Gambia would enhance the robustness of our findings. In addition, it is worth acknowledging that the choice of A/HAR13 as a reference strain collected in 1958 in Egypt for SvA sequences and B/Jali20 as a reference strain collected in 1985 in The Gambia for SvB sequences may have introduced some bias into the variation frequency and accumulation data among SvA and SvB populations [60,62].

In conclusion, while Gambian SvB variants appear to be well adapted to the population and need little further adaptation in order to maintain themselves within the Gambian population, SvA variants exhibit a propensity for diversification, accumulating new mutations. Our findings suggest that geographical factors play a role in driving the diversification and adaptation of Ct strains, highlighting the impact of geospatial differences on Ct evolution. The distinct geospatial distribution of the sequences in which SvA sequences originate from three neighbouring villages in close proximity, while SvB sequences originate from two distinct villages on opposite sides of the River Gambia, provided insights into the village-specific adaptation of SvB strains. Conversely, the proximity of villages with SvA prevalence may contribute to increased circulation of SvA strains among the villages, enhancing the overall diversity and the exchange of variations within the SvA population. Previously, differences in the rate of mutation accumulation in the chromosome of Ct strains was explained by the influence of various factors, including adaptation dynamics, different mutation accumulation and repair speed, geographical specifications, and the influence of mass community-level treatment [15,24, 27, 61, 111,114]. We emphasize that a more extensive investigation in a larger trachoma-endemic population, involving participants over an extended timeframe, is necessary to investigate our speculation suggesting different mutation accumulation rates in the chromosome of ocular Ct strains. Finally, our observations imply that the degree of evolutionary pressure on ocular Ct strains may vary, reflecting the specific fitness of each Ct strain, as manifested in the specific genes experiencing the highest evolutionary pressure in the Gambian SvA compared to SvB variants.

## supplementary material

10.1099/mgen.0.001210 Uncited Supplementary Material 1.

## Abbreviations

Ct: *Chlamydia trachomatis*
ddPCR: droplet digital PCR
DUB: deubiquitinase
EB: elementary body
ENA: European Nucleotide Archive
GTR: Generalized Time Reversible
LGV: lymphogranuloma venereum
MDA: mass drug administration
PZ: plasticity zone
qPCR: quantitative PCR
SNP: single nucleotide polymorphism
SNV: single nucleotide variant
SvA: serovar A
SvB: serovar B
Trp: tryptophan
T3SS: type III secreted proteins
UGT: urogenital
WGS: whole genome sequencing
