# Supplementary material for: Genomic insights into local-scale evolution of ocular Chlamydia trachomatis strains within and between individuals in Gambian trachoma-endemic villages
Source: Microb Genom. 2024 Mar 6;10(3):001210. doi: 10.1099/mgen.0.001210 (PMC10999739; doi:10.1099/mgen.0.001210)
Supplement: Uncited Supplementary Material 1. [file mgen-10-01210-s001.pdf]

Supplementary Data

Genomic insights into local scale evolution of ocular *Chlamydia trachomatis* strains within and between individuals in Gambian trachoma-endemic villages

Ehsan Ghasemian[1*], Nkoyo Faal[2], Harry Pickering[1], Ansumana Sillah[3], Judith Breuer[4], Robin L. Bailey[1], David Mabey[1], Martin J. Holland[1]

[1] Department of Clinical Research, London School of Hygiene & Tropical Medicine, London, United Kingdom

[2] Medical Research Council Unit The Gambia at London School of Hygiene and Tropical Medicine, Banjul, The Gambia

[3] National Eye Health Programme, Ministry of Health, Kanifing, The Gambia

[4] Division of Infection and Immunity, University College London, London, United Kingdom

[*]Correspondence: ehsan.ghasemian@lshtm.ac.uk

Data S1.

Chlamydia trachomatis strains B/HAR36 and B/Tunis864

The London School of Hygiene & Tropical Medicine (LSHTM) trachoma group obtained live stocks and genomic DNA from *Chlamydia trachomatis* (Ct) strains A/2497, A/2497P-, B/HAR36 and C/TW3 via Harlan Caldwell at the Laboratory of Intracellular Parasites, Rocky Mountain Laboratories, National Institute of Allergy and Infectious Diseases, National Institutes of Health, Hamilton, Montana. Comparative genomic investigations on these strains were published by Kari *et al.* [1]. LSHTM provided the Wellcome Trust Sanger Institute (WTSI) genomic DNA from A/2497, B/HAR36 and C/TW3, which underwent full genome sequencing by Next Generation Sequencing and the sequences deposited in the European Nucleotide Archive (ENA) under the following accession numbers (CP002401, ERR189736, and CP006945, respectively). These sequences were included in analyses by Andersson *et al.* [2] and Hadfield *et al.* [3]. At the start of the EU Horizon-2020 funded TracVac project (2017 – 2022, Grant agreement code: 733373) B/HAR36 was selected for large scale culture to represent serovar B genotypes as part of *in vitro* and *in vivo* vaccine studies. Subsequently DNA extracted from these B/HAR36 cultures were subjected to *omp*A chain-termination sequencing (Sanger sequencing). B/HAR36 labelled *omp*A sequence was found to be a 100% match for NCBI accession DQ064280 *omp*A from B/Tunis864 with a lower identity (99%) to NCBI accession DQ064297.1 *omp*A sequence from B/HAR36. *omp*A sequence from whole genome sequence ERR189736 was extracted and was a match for B/Tunis864 (DQ064280). We therefore, obtained original stocks of B/HAR36 and B/Tunis864 from Julius Schachter and Jeanne Moncada at the World Health Organization Collaborating Centre for Reference and Research on Trachoma and Other Chlamydial Infections, Francis I Proctor Foundation for research in Ophthalmology, University of California, San Francisco (UCSF) and subjected these to Whole-Genome Sequencing along with ERR189736 DNA originally supplied to

WTSI. We found that the whole genome sequence initially deposited to the ENA as strain B/HAR36 (ERR189736) was a closer match with the new genome sequence of strain B/Tunis864 with three variations between the sequences. The new B/Tunis864 genome sequence, is now deposited under the ENA accession number ERR12253485. B/HAR36 original stock obtained from UCSF was distinct from B/Tunis864 at the whole genome level with 1,407 variations and the extracted *omp*A sequence was a match (100%) for NCBI DQ064297.1 *omp*A (B/HAR36 *omp*A); the new genome sequence of strain B/HAR36 is now deposited as ERR12253486. Since the full genome sequence ERR189736 initially labelled as B/HAR36 and B/Tunis864 full genome sequence (ERR12253485) represent the same strain genome, the observation that ERR189736 carried B/Tunis864 *omp*A is not explained by *omp*A recombination during laboratory culture as at the time of culture B/HAR36 was not available in the LSHTM laboratories or at the Statens serum Institute, Copenhagen, Denmark where bulk culture was performed as part of TracVac. We therefore infer that there was a labelling mistake on stock that was sequenced to generate ERR189736; correction of metadata for this record was performed and the new genome sequences were deposited in ENA (project accession PRJEB68374).

References

1. Kari, L.; Whitmire, W.M.; Crane, D.D.; Reveneau, N.; Carlson, J.H.; Goheen, M.M.; Peterson, E.M.; Pal, S.; de la Maza, L.M.; Caldwell, H.D. Chlamydia Trachomatis Native Major Outer Membrane Protein Induces Partial Protection in Nonhuman Primates: Implication for a Trachoma Transmission-Blocking Vaccine. *The Journal of Immunology* **2009**, *182*, 8063, doi:10.4049/jimmunol.0804375.
2. Andersson, P.; Harris, S.R.; Smith, H.M.B.S.; Hadfield, J.; O’Neill, C.; Cutcliffe, L.T.; Douglas, F.P.; Asche, L.V.; Mathews, J.D.; Hutton, S.I.; et al. Chlamydia Trachomatis

from Australian Aboriginal People with Trachoma Are Polyphyletic Composed of Multiple Distinctive Lineages. *Nat Commun* **2016**, *7*, 10688, doi:10.1038/ncomms10688.

3. Hadfield, J.; Harris, S.R.; Seth-Smith, H.M.B.; Parmar, S.; Andersson, P.; Giffard, P.M.; Schachter, J.; Moncada, J.; Ellison, L.; Vaulet, M.L.G.; et al. Comprehensive Global Genome Dynamics of Chlamydia Trachomatis Show Ancient Diversification Followed by Contemporary Mixing and Recent Lineage Expansion. *Genome Res* **2017**, *27*, 1220–1229, doi:10.1101/gr.212647.116.

Data S2.

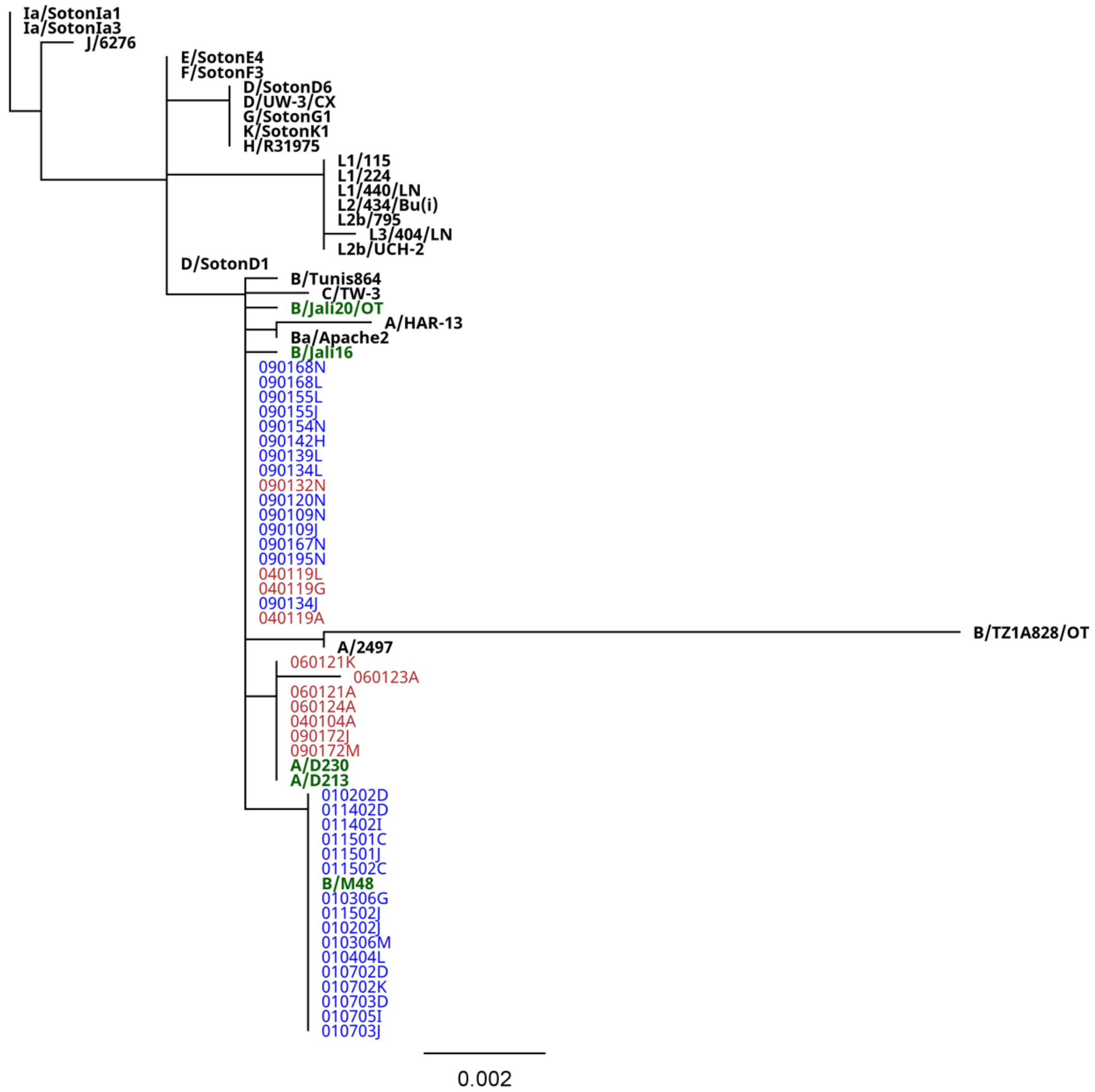

Fig. S1. Phylogenetic analysis of *Chlamydia trachomatis trp*AB genes. The phylogenetic tree encompasses 41 *C. trachomatis* (Ct) positive samples obtained from The Gambia, alongside 29 Ct reference strains. Sequence alignments were generated using MAFFT, and maximum likelihood phylogenies of these aligned sequences were estimated employing the General Time Reversible (GTR) model of evolution with 1000 bootstrap replicates, facilitated by PhyML. The Gambian samples testing positive for Ct serovar A are denoted in red, while those for serovar B are indicated in blue. All reference strains are highlighted in bold, with strains originating from The Gambia represented in green. The scale bar indicates evolutionary distance.

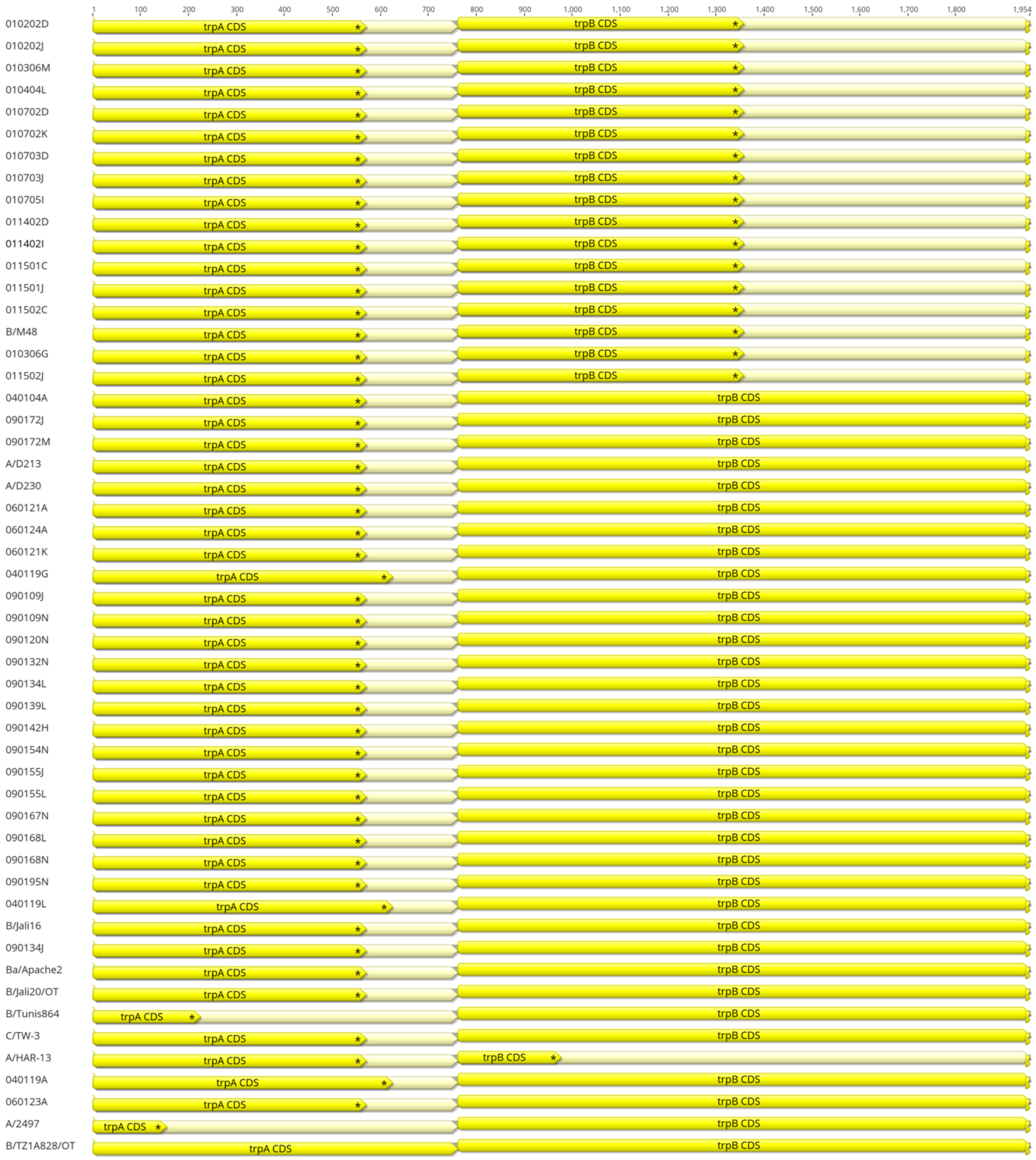

Fig. S2. Comparative alignment of *Chlamydia trachomatis trp*AB Genes. Alignment of *trp*AB genes from *C. trachomatis* (Ct) sequences sourced from the Gambia and Ct ocular reference strains was conducted using MAFFT (version v7.490). The asterisk in the annotation indicates the termination of the coding sequence.

Table S1. Recorded baseline demographics, trachoma grades, *Chlamydia trachomatis ompA* and plasmid type, and infection loads for study samples.

	Sample ID	Sex	Age	Trachoma grade	pORF2 load	*omc*B load	*omp*A genotype	Plasmid genotype
**Village 1**								
	010202_D	F	5	TF	79.4	12.5	B	B
	010202_J	F	5	TF	121.5	17.1	B	B
	010306_G	F	5	TF	22992.1	3071.4	B	B
	010306_M	F	5	TF	1107.6	184.6	B	B
	010404_L	F	10	TF	679	37.3	B	B
	010702_D	F	7	TF	318.6	54.4	B	B
	010702_K	F	7	TF	312.4	58.5	B	B
	010703_D	F	4	TF	9736.2	1388.1	B	B
	010703_J	F	4	TF	4619.7	837.1	B	B
	010705_I	M	10	Normal	525.8	68.8	B	B
	011402_D	F	10	TF	17460	3063.6	B	B
	011402_I	F	10	TF	5074.9	857.8	B	B
	011501_C	F	5	TF	2503.1	303.5	B	B
	011501_J	F	5	TF	6200.4	2798.9	B	B
	011502_C	F	4	TF	1214.3	279.8	B	B
	011502_J	F	4	TF	767.8	106.4	B	B
**Village 4**								
	040104_A	M	6	TF	1646	249.3	A	A
	040119_A	M	13	TF	137.8	27	A	A
	040119_G	M	13	TF	250	31.8	A	A
	040119_L	M	13	TF	157.1	22.6	A	A
**Village 6**								
	060121_A	M	8	TF	7516.9	653.7	A	A
	060121_K	M	8	Normal	761	63.6	A	A
	060123_A	M	7	TF	1830.2	216	A	A
	060124_A	F	8	TF	287.2	24.7	A	A
**Village 9**								
	090109_J	M	15	TF	2556.8	639.2	B	B
	090109_N	M	15	Normal	997.5	176.8	B	B
	090120_N	M	9	Normal	342.5	69.3	B	B
	090132_N	M	13	TF	147.5	29.5	A	A
	090134_J	M	9	TF	235	44.9	B	B
	090134_L	M	9	TF	802.1	183.8	B	B
	090139_L	M	9	TF	182.5	34.5	B	B
	090142_H	M	13	TF	116.4	27	B	A
	090154_N	M	11	Normal	307.5	69	B	B
	090155_J	M	9	TF	192.1	41.6	B	B
	090155_L	M	9	TF	2424.5	553.1	B	B
	090167_N	M	9	Normal	153.7	28.8	B	B
	090168_L	M	10	TF	1780	500.8	B	B
	090168_N	M	10	Normal	297.9	55.6	B	B
	090172_J	M	11	TF	5449.3	1052	A	A
	090172_M	M	11	TF	4311.9	815.2	A	A
	090195_N	M	8	TF	68.5	37.5	B	B

Table S2. *Chlamydia trachomatis* whole-genome sequencing data and quality information for study samples.

	Sample ID	Number of raw read pairs	read pairs after trimming, qualiy score filtering, and merging	Per base sequence quality	Average quality per read	% GC	% per base N content	Read mean length (bp)	Number of read paired classified as *Chlamydia trachomatis*	Mean coverag of *Chlamydia trachomatis* reference genome	Std Dev (mean coverag of *Chlamydia trachomatis* reference genome)	Ref-seq (%)	Confidence mean	Chromosome length (bp)
**Village 1**														
	010202_D	3,607,364	1,413,099	>28	38	40	0	153	122,173	21.3	14	99.4	37.5	1,044,421
	010202_J	3,608,774	1,191,509	>28	38	43	0	151	356,920	53.6	28	99.6	37.6	1,044,762
	010306_G	4,041,512	1,374,631	>28	38	41	0	170	914,801	142	54.5	99.6	37.5	1,044,442
	010306_M	3,859,352	1,480,843	>28	38	41	0	162	238,390	41.7	25.5	99.6	37.6	1,044,424
	010404_L	3,495,140	1,114,702	>28	37	41	0	163	545,362	85.7	35.3	99.6	37.4	1,044,484
	010702_D	2,476,052	677,749	>28	38	41	0	168	396,714	65.8	24.8	99.6	37	1,044,429
	010702_K	3,622,540	1,328,892	>28	37	43	0	170	452,429	80.9	48.3	99.6	37.5	1,044,503
	010703_D	3,304,646	937,981	>28	37	41	0	172	636,957	105.2	40.6	99.6	37	1,044,482
	010703_J	2,511,736	724,754	>28	37	41	0	163	483,233	80.7	31.1	99.6	37	1,044,502
	010705_I	3,151,364	1,022,956	>28	37	41	0	172	591,994	94.2	36.1	99.6	37.3	1,044,419
	011402_D	4,590,522	1,281,634	>28	37	41	0	172	876,389	146.5	54.3	99.6	36.9	1,044,511
	011402_I	4,529,892	1,279,478	>28	37	41	0	172	887,090	148.7	54.1	99.6	37	1,044,505
	011501_C	3,573,360	1,156,467	>28	37	41	0	163	743,013	117.1	45.8	99.6	37.4	1,044,480
	011501_J	6,688,138	1,913,661	>28	37	41	0	172	1,334,258	222.4	80.2	99.6	37	1,044,512
	011502_C	3,913,356	1,463,596	>28	38	42	0	179	1,196,075	210.1	115.6	99.6	37.6	1,044,534
	011502_J	3,076,780	874,013	>28	37	42	0	169	432,783	71.7	29.3	99.6	37.1	1,044,455
**Village 4**														
	040104_A	2,337,432	611,793	>28	37	42	0	173	347,444	58.6	24.1	99.6	36.9	1,044,521
	040119_A	2,457,774	637,256	>28	37	43	0	169	246,632	41.4	18.2	99.5	36.9	1,044,531
	040119_G	3,684,418	1,313,457	>28	38	44	0	166	406,104	68	32.3	99.5	37.8	1,044,729
	040119_L	3,259,084	1,031,894	>28	38	43	0	156	327,882	51.6	22.8	99.5	37.4	1,044,655
**Village 6**														
	060121_A	3,431,668	1,149,974	>28	38	41	0	161	723,676	113.9	47.2	98.1	37.4	1,044,561
	060121_K	2,517,680	668,524	>28	37	42	0	166	273,864	44.4	21.4	98.1	37.2	1,044,606

	060123_A	4,191,512	1,571,119	>28	38	41	0	149	908,894	132.6	64	98	37.9	1,044,616
	060124_A	3,342,888	904,124	>28	37	42	0	167	327,738	54.3	24	98.1	37.1	1,044,548
**Village 9**														
	090109_J	4,799,546	2,171,961	>28	40	42	0	199	1,332,150	270.9	154.2	99.6	40	1,044,476
	090109_N	3,287,146	1,558,956	>28	40	43	0	203	901,921	178	67	99.6	40.3	1,044,457
	090120_N	3,638,672	1,748,766	>28	41	42	0	170	951,485	165.4	64.7	99.6	40.4	1,044,425
	090132_N	3,881,366	1,804,625	>28	40	44	0	205	708,070	144.3	56.9	99.6	40.2	1,044,562
	090134_J	3,785,094	1,446,284	>28	38	43	0	157	108,097	19.1	17.1	96.2	37.5	1,044,404
	090134_L	2,389,658	647,184	>28	37	42	0	169	325,673	54.2	22	99.6	37	1,044,436
	090139_L	3,614,836	1,695,248	>28	40	43	0	204	837,136	164.1	63.2	99.6	40.2	1,044,451
	090142_H	3,377,450	1,158,843	>28	38	43	0	174	406,393	70.5	30	99.6	37.6	1,044,567
	090154_N	3,510,798	1,642,701	>28	40	43	0	214	891,817	187.1	69.4	99.6	40.1	1,044,440
	090155_J	3,359,078	1,289,653	>28	38	41	0	164	400,653	70.3	43.2	99.6	37.6	1,044,488
	090155_L	2,692,100	765,780	>28	37	42	0	171	507,780	84.7	33	99.6	37	1,044,419
	090167_N	3,276,578	1,519,348	>28	40	44	0	205	495,844	102.1	44.4	99.6	40.1	1,044,441
	090168_L	3,649,316	1,225,599	>28	38	42	0	181	683,884	121.7	43.9	99.6	37.4	1,044,424
	090168_N	3,598,198	1,717,357	>28	40	42	0	195	1,163,730	227.5	81.3	99.6	40.2	1,044,441
	090172_J	3,888,716	1,324,270	>28	38	41	0	180	763,165	134.6	49	99.6	37.4	1,044,651
	090172_M	3,442,574	1,051,627	>28	37	41	0	168	697,247	113.9	43.2	99.6	37.2	1,044,513
	090195_N	3,698,022	1,765,573	>28	40	42	0	207	1,260,658	255.5	94.6	99.6	40.2	1,044,432

Table S3. List of accession numbers and metadata for all *Chlamydia trachomatis* reference strains used in this study.

Isolate name	Lineage	Genotype	Country	Year	Source	Genome-ERR	Genome-ERS	Genome-accession number	Genome-reference	Plasmid-ERR	Plasmid-ERS	Plasmid-accession number	Plasmid-reference
**A/2497**	ocular	A	Tanzania	2000	ocular	-	-	FM872306	Harris et al. 2012	-	-	NC_020550	Clarke and Seth-Smith. 2008
**A/D213**	ocular	A	Gambia	2001	ocular	ERR175652	ERS177838	-	Andersson et al. 2016	ERR175652	ERS177838	-	-
**A/D230**	ocular	A	Gambia	2001	ocular	ERR111554	ERS075177	-	Hadfield et al. 2017	ERR111554	ERS075177	-	-
**A/HAR13**	ocular	A	Egypt	1958	conjunctiva		-	CP000051	Carlson 2005	-	-	NC_007430	Carlson et al., 2005
**B/Jali16**	ocular	B	Gambia	1985	ocular	ERR189738	ERS153015	-	Hadfield et al. 2017	ERR189738	ERS153015	-	-
**B/Jali20**	ocular	B	Gambia	1985	ocular	-	-	FM872308	Seth-Smith et al 2009	-	-	NC_012629	Seth-Smith et al. 2009
**B/TZ1A828**	ocular	B	Tanzania	1998	ocular	-	-	FM872307	Seth-Smith et al 2009	-	-	FM865437	Seth-Smith et al. 2009
**B/M48**	ocular	B	Gambia	2007	ocular	ERR175631	ERS177817	-	Hadfield et al. 2017	ERR175631	ERS177817	-	-
**B/Tunis864**	ocular	B	Tunisia	1976	ocular	ERR12253485	ERS16770978	-	This study	ERR12253485	ERS16770978	-	This study
**Ba/Apache2**	ocular	Ba	USA	1960	ocular	ERR140762	ERS095032	-	Andersson et al. 2016	ERR140762	ERS095032	-	Andersson et al. 2016
**C/TW3**	ocular	C	Taiwan	1959	ocular	ERR558499	ERS177778	-	Andersson et al. 2016	-	-	NC_023057	Borges et al. 2014
**D/SotonD1**	genital	D	UK	2009	endocervix	ERR027327	ERS008761	-	Harris et al. 2012	-	-	HE603229	Harris et al. 2012
**D/SotonD6**	genital	D	UK	2009	endocervix	ERR027328	ERS008762	-	Harris et al. 2012	-	-	NC_020959	Harris et al. 2012
**D/UW3**	genital	D	USA	1965	endocervix	-	-	AE001273	Stephens Science 1998	-	-	-	-
**E/SotonE4**	genital	E	UK	2009	endocervix	ERR026551	ERS013791	-	Harris et al. 2012	-	-	NC_020987	Harris et al. 2012
**F/SotonF3**	genital	F	UK	2009	endocervix	ERR027330	ERS008764	-	Harris et al. 2012	-	-	HE603234	Harris et al. 2012
**G/SotonG1**	genital	G	UK	2009	endocervix	ERR026560	ERS013800	-	Harris et al. 2012	-	-	NC_020961	Harris et al. 2012
**H/R31975**	genital	H	Russia	2010	endocervix	ERR111606	ERS082923	-	Andersson et al. 2016	ERR111606	ERS082923	-	Andersson et al. 2016
**Ia/SotonIa1**	genital	I-Ia	UK	2009	endocervix	ERR026555	ERS013804	-	Harris et al. 2012	-	-	NC_020962	Harris et al. 2012
**Ia/SotonIa3**	genital	I-Ia	UK	2009	endocervix	ERR026565	ERS013805	-	Harris et al. 2012	-	-	NC_020989	Harris et al. 2012

**J/6276**	genital	J	-	-	endocervix	-	-	ABYD01000001	Suchland et al. 2008	-	-	-	-
**K/SotonK1**	genital	K	UK	2009	endocervix	ERR026559	ERS013799	-	Harris et al. 2012	-	-	HE603238	Harris et al. 2012
**L1/115**	LGV	L1	South Africa	1986	urethra	ERR008593	ERS001411	-	Harris et al. 2012	-	-	NC_020951	Harris et al. 2012
**L1/224**	LGV	L1	USA	1968	lymph node	ERR008595	ERS001396	-	Harris et al. 2012	-	-	NC_020952	Harris et al. 2012
**L1/440**	LGV	L1	South Africa	1986	urethra	ERR211058	ERS161108	-	Harris et al. 2012	-	-	NC_010029	Hatt. 1999
**L2/434/Bu**	LGV	L2	USA	1968	bubo	-	-	AM884176	Thomson et al 2008	-	-	NZ_AM886278	Thomson et al., 2008
**L2b/795**	LGV	L2b	France	2004	rectum	ERR008586	ERS001409	-	Harris et al. 2012	-	-	NC_020983	Harris et al. 2012
**L2b/UCH2**	LGV	L2b	UK	-	proctitis	ERR008587	ERS001405	-	Harris et al. 2012	-	-	NC_020956	Harris et al. 2012
**L3/404**	LGV	L3	USA	1967	lymph node	ERR008583	ERS001416	-	Harris et al. 2012	-	-	NC_020957	Harris et al. 2012

Table S4. Genes with the highest (Top 1%) number of single nucleotide variant (SNV) accumulation in SvA and SvB sequences.

CDS	Position (Min)	Position (Max)	SNV
***Chlamydia trachomatis* SvA**			
membrane protein CDS	68,149	69,252	9
type III secretion system effector protein CDS	94,989	96,671	15
ABC transporter substrate-binding protein CDS	156,524	157,804	8
DNA ligaseA CDS	164,295	166,286	8
hypothetical protein CDS	166,384	170,733	19
YitT family protein CDS	251,627	252,523	8
inclusion protein (inc) A family CDS	324,073	325,764	11
HEAT repeat domain-containing protein CDS	403,603	403,596	8
hypothetical protein CDS	412,199	412,735	9
translocated actin recruiting phosphoprotein (tarp) CDS	533,311	536,631	14
hypothetical protein CDS	709,788	711,743	32
SufD family Fe-S cluster assembly protein CDS	790,296	791,483	18
hypothetical protein CDS	847,887	849,233	8
deubiquitinase CDS	1,026,820	1,028,076	15
***Chlamydia trachomatis* SvB**			
translation initiation factor IF-2 (infB) CDS	110,591	113,269	4
hypothetical protein CDS	166,347	170,696	5
inclusion protein (inc) A family CDS	256,946	257,534	5
translocated actin recruiting phosphoprotein (tarp) CDS	533,228	536,524	4
rpsC CDS	595,062	595,736	5
UvrD-helicase domain-containing protein CDS	689,703	691,607	4
DNA primase (dnaG) CDS	931,093	932,880	4
polymorphic membrane protein (pmp) D CDS	953,168	957,763	6

Table S5. Genes with the highest (Top 1%) number of nonsynonymous to synonymous single nucleotide variant (SNV) among SvA and SvB sequences.

**CDS**	**Position (Min)**	**Position (Max)**
***Chlamydia trachomatis* SvA**		
membrane protein CDS	68,149	69,252
npt1 CDS	77,379	78,959
type III secretion system effector protein CDS	94,989	96,671
YitT family protein CDS	251,627	252,523
hypothetical protein CDS	274,398	275,593
hypothetical protein CDS	281,737	282,087
biotin transporter BioY CDS	412,795	413,385
secD CDS	521,627	525,828
translocated actin recruiting phosphoprotein (tarp) CDS	533,311	536,631
***Chlamydia trachomatis* SvB**		
infB CDS	110591	113269
hypothetical protein CDS	166,347	170,696
DUF3491 domain-containing protein CDS	191,189	194,041
trpB CDS	195,097	196,275
CTP synthase CDS	206,611	208,203
MFS transporter CDS	262,582	265,368
translocated actin recruiting phosphoprotein (tarp)	533228	536524
rpsC CDS	595,062	595,736
UvrD-helicase domain-containing pr CDS	689703	691607
RNA polymerase factor sigma-54 CDS	691,611	692,921
FAD-dependent thymidylate synthase CDS	720,998	722,587
dnaG CDS	931093	932880
glmM CDS	959,813	961,189

Table S6. Genes with the highest (Top 1%) number of single nucleotide polymorphism accumulation (SNP) over a short timeframe (4-20 weeks).

***Chlamydia trachomatis* SvA/SvB**

CDS	Position (Min)	Position (Max)	SNP
isoleucyl-tRNA synthetase CDS	22,026	25,136	4
class I SAM-dependent methyltransferase CDS	151,080	151,883	6
phospholipase D-like domain-containing protein CDS	181,988	183,184	3
hypothetical protein CDS	185,044	185,545	3
hypothetical protein CDS	197,792	197,962	3
small cysteine-rich outer membrane protein CDS	516,100	516, 366	3
translocated actin recruiting phosphoprotein (tarp) CDS	533,228	536,524	5
deubiquitinase CDS	1,026,714	1,027,970	3
